# Heat-Shock Promoters: Targets for Evolution by *P* Transposable Elements in *Drosophila*

**DOI:** 10.1371/journal.pgen.0020165

**Published:** 2006-10-06

**Authors:** Jean-Claude Walser, Bing Chen, Martin E Feder

**Affiliations:** 1 Department of Organismal Biology and Anatomy, The College, The University of Chicago, Chicago, Illinois, United States of America; 2 The Committees on Evolutionary Biology, Genetics, and Molecular Medicine, The College, The University of Chicago, Chicago, Illinois, United States of America; Fred Hutchinson Cancer Research Center, United States of America

## Abstract

Transposable elements are potent agents of genomic change during evolution, but require access to chromatin for insertion—and not all genes provide equivalent access. To test whether the regulatory features of heat-shock genes render their proximal promoters especially susceptible to the insertion of transposable elements in nature, we conducted an unbiased screen of the proximal promoters of 18 heat-shock genes in 48 natural populations of *Drosophila*. More than 200 distinctive transposable elements had inserted into these promoters; greater than 96% are *P* elements. By contrast, few or no *P* element insertions segregate in natural populations in a “negative control” set of proximal promoters lacking the distinctive regulatory features of heat-shock genes. *P* element transpositions into these same genes during laboratory mutagenesis recapitulate these findings. The natural *P* element insertions cluster in specific sites in the promoters, with up to eight populations exhibiting *P* element insertions at the same position; laboratory insertions are into similar sites. By contrast, a “positive control” set of promoters resembling heat-shock promoters in regulatory features harbors few *P* element insertions in nature, but many insertions after experimental transposition in the laboratory. We conclude that the distinctive regulatory features that typify heat-shock genes (in *Drosophila*) are especially prone to mutagenesis via *P* elements in nature. Thus in nature, *P* elements create significant and distinctive variation in heat-shock genes, upon which evolutionary processes may act.

## Synopsis

Transposable elements can be a major source of evolutionary change. Their insertion can directly affect the genes into, or next to, which they insert. To insert, however, they must first gain access to the host gene. The authors reasoned that, because the DNA in the promoters (i.e., regulatory regions) of heat-shock genes is unusually accessible, these genes might harbor many transposable elements. With a technique that can detect any insertion into a gene, they discovered more than 200 distinctive transposable elements in the promoter regions of heat-shock genes in fruit flies from the wild—but few or none in the promoter regions of more typical genes. Surprisingly, out of the one hundred kinds of transposable elements in fruit flies, almost all were *P* elements. *P* elements are remarkable because they invaded the fruit fly genome only during the last century. These findings imply that the combination of accessible DNA and the recent invasion of *P* elements have left a distinctive imprint on the promoters of heat-shock genes.

## Introduction

Genes may vary in evolvability for many reasons, including physical susceptibility to mutagenesis. Here we show that a class of genes with distinctive physical features—heat-shock genes—is remarkably prone to mutagenesis by insertion of a specific transposable element (TE), the *P* element of *Drosophila*.

TEs are mobile, repetitive DNA sequences and a structurally dynamic component of genomes [1]. TEs can cause gene and chromosome evolution in numerous ways, including insertional mutagenesis, retroposition, conveyance of regulatory elements to novel sites, and service as pivotal sites for ectopic recombination, and thus chromosomal rearrangements and gene duplication. For such evolution to occur, however, TEs must first insert into chromatin, which in turn requires that the target site be accessible to the transpositional machinery [2]. Indeed, insertion of *Drosophila P* elements, among the best-studied of TEs [3], into specific sites is associated with features of local chromatin architecture such as DNase I hypersensitivity, location in 5′-flanking sequence, presence of pre-existing TEs, and physical structure, but only weakly with insertion sites' nucleotide sequence (e.g., [4–6]). These features vary widely and frequently throughout genomes [7], which is consistent with the irregular, but repeated, occurrence of TEs. Entire classes of genes also vary in TE frequency—and hence potentially evolvability via transposition—in laboratory studies [8], but for natural populations, neither the mechanistic basis for this variation nor its relevance for evolvability is clear.

In such experimental work with *Drosophila,* heat-shock genes (e.g., the major heat-shock gene *Hsp70*) stand out as a class receiving numerous TE insertions [8–10]. (By “gene,” we intend both the transcribed sequence and associated non-transcribed regulatory sequence.) This distinction is not unexpected from two perspectives. First, the local chromatin architecture of heat-shock proximal promoters is peculiar, incorporating constitutively decondensed chromatin and nucleosome-free regions [11,12], and constitutive engagement of the transcriptional machinery. In addition to the 5′ location of these promoters, such features should predispose these regions to TE insertion (see above). Second, TEs segregate at high frequency in natural populations in the 5′-flanking regions of the five genomic copies of *Hsp70* [13–17]. This finding is remarkable given that TEs typically are at low allelic frequency in the *Drosophila* genome, presumably because they are deleterious [18–20]. The *Hsp70* intragenic TEs are seemingly adaptive, exhibiting repeatable demographic variation in allelic frequency along natural thermal gradients and beneficial impacts on *Hsp70* expression and components of fitness [14–17,21]. Nonetheless, TEs constitute 22% of the *Drosophila* genome [22] and are numerous (more than 6,000 elements) [23]. Thus TE insertions in heat-shock genes could simply be a manifestation of general patterns because TEs are common in the *Drosophila* genome, rather than indicative of a specific insertion susceptibility and/or adaptive role. To distinguish between these possibilities, we carried out an unbiased screen with both negative and positive controls. Our working hypotheses were as follows:

First, because TE insertion can be mutagenic, naturally occurring transposition into *Hsp70* genes could simply reflect that these are multicopy genes [24] and functionally redundant, and thus permit insertional mutagenesis of one to two copies. If so, then TEs occurring in proximal promoter regions should be restricted to multicopy genes like *Hsp70* and not widespread in the “heat-shock genome” of natural populations, typically comprising single-copy genes.

Second, if as a class, heat-shock genes are especially susceptible to TE insertion in their proximal promoter region, the “heat-shock genome” of natural *Drosophila* populations should harbor numerous TEs in this region. Accordingly, we screened for TEs in the proximal promoters of 18 heat-shock genes other than *Hsp70*. This set of genes represents the prototypical heat-shock genes and cognates in Drosophila melanogaster other than *Hsp70* (Gene Set I in Table 1).

**Table 1:**
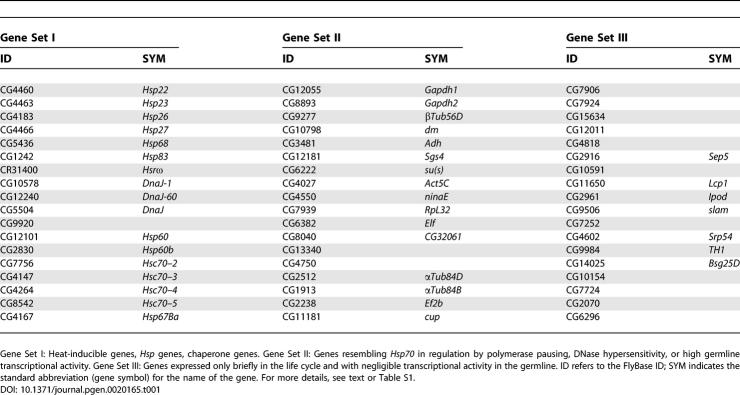
Genes Other Than *Hsp70* Screened for Transposable Element Insertions in 5′-Flanking Sequence

Third, if heat-shock genes' peculiar chromatin architecture and its correlates (see above) predispose the heat-shock genome to TE insertion, then the proximal promoter regions of other genes in the *Drosophila* genome sharing some or all of these features should likewise harbor numerous TEs in natural populations. Accordingly, we screened for TEs in 18 non–heat-shock genes resembling heat-shock genes in relevant features (Gene Set II in Table 1).

Finally, if heat-shock genes' chromatin architecture and its correlates predispose the heat-shock genome to TE insertion, then in natural populations, genes *dissimilar* to heat-shock genes should *less* frequently harbor TEs in their proximal promoter regions. Accordingly, we screened for TEs in the proximal promoters of a “negative control” set of 18 such genes (Gene Set III in Table 1).

Relevant to all working hypotheses is that a TE in a gene will signify both that the TE has successfully inserted and that the TE has not (yet) been eliminated. Remobilization of TEs, their mutagenesis, and negative selection may all affect TEs' presence at a specific site.

Such screens pose a substantial analytical challenge. Only a single D. melanogaster genome has presently been sequenced, and that for an isogenized laboratory strain [25]. Although the sequenced genome is typical of wild D. melanogaster with respect to many TEs, it is intentionally dissimilar with respect to others [26].Moreover, an isogenized strain obviously cannot represent variability present in natural populations. Furthermore, most attempts to characterize the “transposome” of natural *Drosophila* populations, whether experimentally or in silico, are sequence-based; i.e., they rely on the distinctive canonical sequences of the various TEs for TE recognition and subsequent identification of the gene (or intergenic region) in which TEs have inserted. These methods range from genomic Southern blots to TE-specific PCR to TE display to bioinformatics searches. Our objective, by contrast, is to ascertain how often specified gene regions contain TEs. Given that each region to be screened might contain one of more than 120 different TE families in *Drosophila* [23,26], a sequence-based screen specific for each possible element in numerous genes and populations would be prohibitively laborious. Furthermore, our region of interest (proximal promoter) is non-coding, which may frustrate simple PCR-based screens when highly variable. For these reasons we have exploited universal fast walking (UFW) [27,28], a method that can report TEs, not by their sequence, but by the size polymorphisms they create.

**Figure 1:**
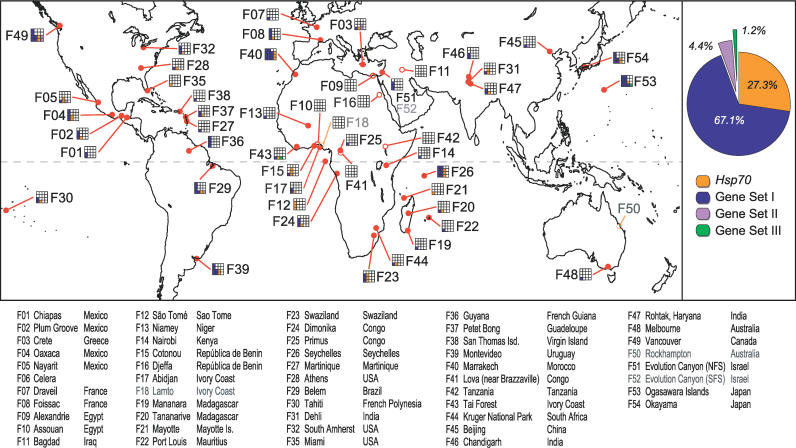
Geographic Origins of D. melanogaster Populations Screened in This Study Screens revealed zero to 14 *P* elements per population (indicated by the number of squares), distinctive by insertion location, in the proximal promoter regions of genes examined (Table 1). Colors of squares correspond to gene set (see Introduction). Inset: Percentages of distinctive *P* elements discovered in *Hsp70* genes and each of the three gene sets screened. A total of 161 *P* element insertions (the ten *P* elements in the coding sequence and the five non–*P* element insertions are not included in the figure). These tallies potentially under-report the actual number of *P* elements; see Results. F06 (Celera) is the strain whose genome has been sequenced [25] and is the reference strain for the present study. Populations F18, F50, and F52 (in light gray text) were removed from the analysis after screens failed for multiple genes and primer sets.

Here we demonstrate, by applying this technique to a screen of 48 natural *Drosophila* populations from around the world (Figure 1), that heat-shock genes as a class are a distinctive and repeatable natural target for TE insertion, as is predictable from the distinctive characteristic features of these promoters. Remarkably, of the many active TE families that might target heat-shock genes, the vast majority of the naturally occurring TEs that we discovered are *P* elements, notorious for their recent invasion of the D. melanogaster genome [29–31]. Accordingly, we conclude that the proximal promoters of heat-shock genes in *Drosophila* are especially conducive to transposition of *P* elements in nature, which creates significant variation upon which evolutionary processes may act. Furthermore, dissimilarities between frequencies of naturally occurring and experimental *P* element transpositions into the various classes of promoters imply that weakened purifying selection and/or positive selection may contribute to the persistence of *P* elements in natural populations—a suggestion that invites future testing.

## Results

### Summary Findings and Methodology

**Figure 2:**
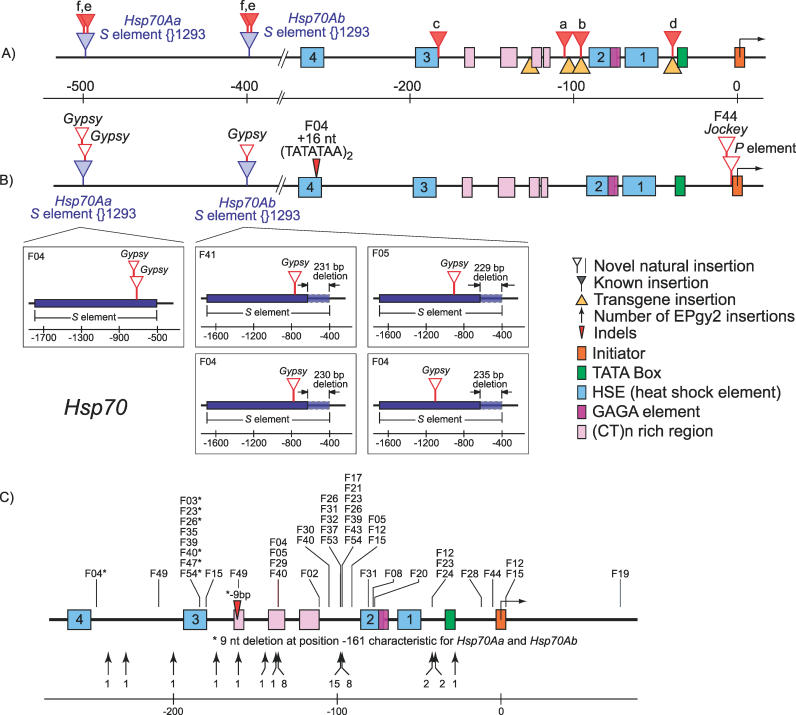
Locations of TEs Integrating into the Proximal Promoters of *Hsp70* Genes Six nearly identical *Hsp70* genes are present in the sequenced *Drosophila* genome, but only five copies in natural populations. The locations of selected promoter elements and sites are indicated for all copies. (A) Previously discovered TEs and experimental transpositions relative to the conserved *Hsp70* sequence. a, *Jockey* element in *Hsp70Ba* [16]; b, c, and d, *P* elements in *Hsp70Ba* [14,15,21]. An *S* element is present between the oppositely oriented paralogs *Hsp70Aa* and *Hsp70Bb* [13], and is represented twice, corresponding to its location relative to each paralog, as are the *HMS Beagle* (e) [16] and “*56H8*” (f) [88] elements inserted within it. Triangles below the line indicate transgene insertion sites (FlyBase; http://flybase.bio.indiana.edu). (B) and (C) Bottom: newly discovered TEs, with the natural population in which they were discovered (F01–F54, exclusive of F06) indicated for each. (B) TEs other than *P* elements. Four are *Gypsy* elements that have integrated into the *S* element in specific populations, the fifth is a *Gypsy* that has inserted into a *Gypsy,* and the sixth is a *Jockey* that has inserted into a *P* element. The *Gypsy*s are arbitrarily plotted relative to *Hsp70Ab* and *Hsp70Aa,* respectively. (C) Natural *P* elements in *Hsp70*. The arrows indicating the number of independent EPgy2 insertion sites recently described by Shilova et al. [10]. Except for the *Gypsy*s, TEs were not mapped to a specific *Hsp70* gene. Insertion sites localized within the *Hsp70* region were all established by sequencing. For population codes, see Figure 1.

**Figure 3:**
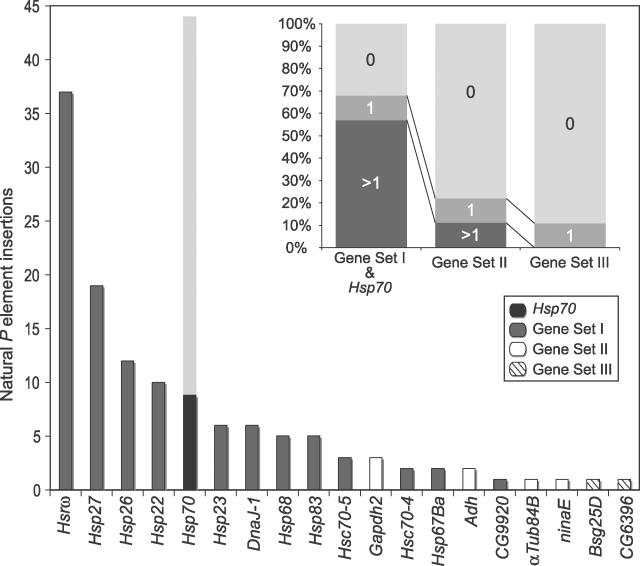
Number of Natural *P* Element Insertions (161 Total) Distinctive by Population and Location into the “Proximal Promoter Region” of Each of the Screened Genes (Table 1) Genes without any such insertions are not represented in the main figure. These tallies and estimates are conservative in three ways: (1) *P* elements inserting within 1,000 bp of the transcription start site of *Bsg25D* and *CG6396* are included although they actually insert into neighboring genes (see Figure 4); (2) The tally for *Hsp70* excludes non–*P* elements and those previously discovered (Figure 2), and divides the remaining total (44, light gray bar in background) by five, the *Hsp70* copy number for natural populations [17]; and (3) Re-screening of a subset of insertions implies an underestimation of the tally at the 161 *P* insertion sites (see Results and Figure 7). Inset: frequencies of genes in each Gene Set (I, including *Hsp70,* II, and III) in which 0, 1, or >1 *P* elements had inserted.

**Figure 4:**
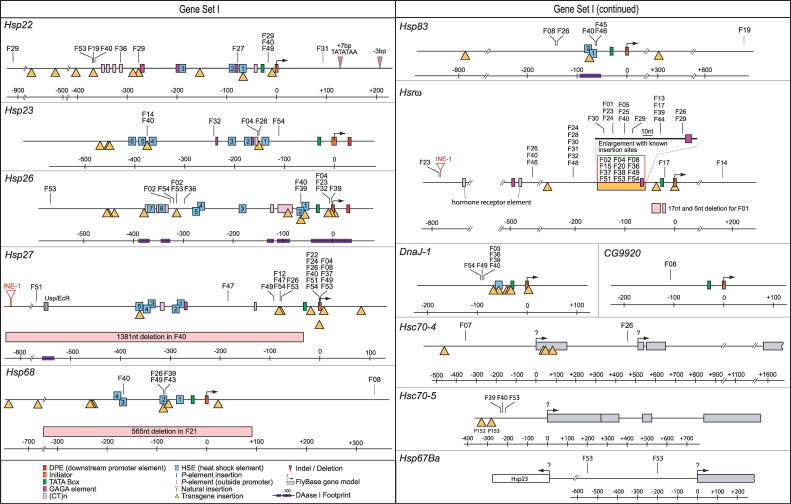
Locations of *P* Elements Integrating into the Proximal Promoters of Heat-Shock Genes Other than *Hsp70* (Gene Set I) Data are plotted as in Figure 2 except as follows: F-numbers in columns refer to natural populations with transposons integrating at identical sites. Primer sets used in the screens amplified regions 3′ to transcription start site of different length; *P* elements discovered upstream of the initiator are plotted (pale), but not included in comparative analyses (i.e., in *Hsp22* in population F31, *Hsp68* in F05, and in *Hsrω* in F14). In *Hsrω*, numerous *P* elements were discovered in one region (box); a randomly chosen subset of these were localized (by sequencing) within that region (see enlargement). Several putative deletions were also discovered, and are plotted. For population codes see Figure 1. Table S1 provides additional information about Gene Set I and relevant sources for the organization of promoter regions.

**Figure 5:**
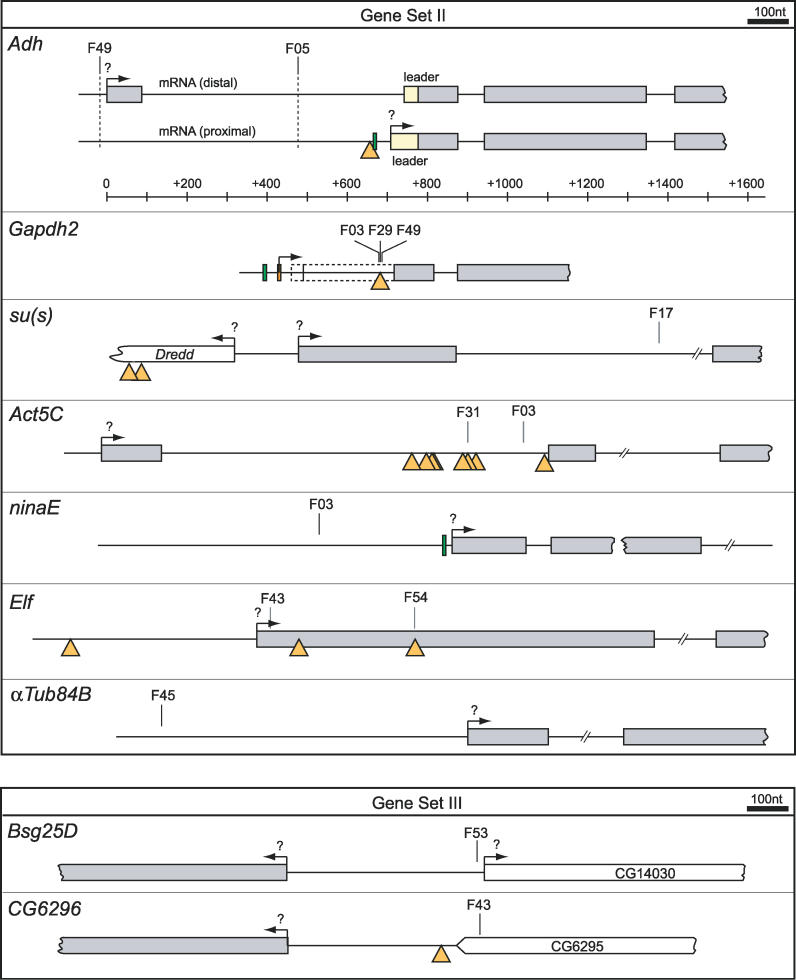
Locations of *P* Elements Integrating into the Proximal Promoters of Non–Heat-Shock Genes Resembling Heat-Shock Genes in Relevant Features of Their Proximal Promoters (Gene Set II), and in Genes Dissimilar to Heat-Shock Genes (Gene Set III) Data are plotted as in Figure 2 except as follows: Primer sets used in the screens amplified regions 3′ to transcription start site of different length; *P* elements discovered upstream of the initiator are plotted (pale), but not included in comparative analyses (i.e., in *su(s)* in population F17, in *Act5C* in F03 and F31, and in *Elf* in F43 and F54). Note that in Gene Set III, the two *P* elements discovered are not clearly associated with their focal genes, integrating into or just upstream of genes neighboring the focal genes. For population codes, see Figure 1. Table 1 provides additional information about the gene sets.

The UFW screen revealed numerous differences in amplicon size between the reference strain (Celera, F06) and the natural populations (see exemplary gel images in [28]). These polymorphisms were characterized by sequencing and/or TE-specific PCR. A total of 97% were insertions of *P* elements into the proximal promoters of the surveyed genes, with the balance *jockey* and *gypsy* elements (Figure 2). In fact, 19 (35%) of all investigated promoters (*N*_genes_ = 55) had at least one *P* element insertion in the proximal promoter region in one or more of the populations investigated (*N*_populations_ = 48; Figures 1 and 3). Many of these insertions are into identical sites in different populations (Figures 2, 4, and 5). Most (42 of 48) populations had a *P* element insertion in at least one investigated gene.

These findings prompt two methodological concerns, which are unwarranted. First, insertions common to multiple populations could be shared by descent, and hence tallying them as independent would overestimate insertion events; we exclude this possibility below. Second, the high proportion of *P* elements could stem from oversensitivity of the UFW screen to *P* elements. To address this concern, we re-screened a subset of genes known to contain both *P* elements and other TEs (the *Hsp70* genes in each natural population) with a technique known to detect all these TEs. The re-screening used a reliable PCR with one primer complementary to *Hsp70* and the other to each of the six TEs common in the Celera strain *(roo, 1360, 297, Jockey, I,* and *Gypsy)* [26]. This procedure re-detected each *Jockey* and *Gypsy* previously implicated by UFW in *Hsp70,* but revealed no additional TEs in any of the other genes. Two genes from each gene set (Gene Sets I–III) were likewise re-screened for four populations (F04, F40, F53, and F54), with the result entirely consistent with the previous screens. These results affirm that the method is universal, detects TEs when present, and does not favor *P* elements.

### Abundance of Transposon Insertions in the Three Gene Sets

The UFW- and TE-specific PCR screens together initially detected 177 TE insertion sites (containing 171 *P* elements; Figures 2, 4, and 5), one *Jockey* inserted in a *P* element, and five *Gypsy* (one a *Gypsy* inserted into a *Gypsy;* Figure 2) in the 55 genes surveyed (Table 1) in the 48 natural *Drosophila* populations (Figure 1). Because the screens intentionally focused on the proximal promoter region of each surveyed gene, 167 of these insertion sites (containing 161 *P* elements, the *Jockey,* and the *Gypsy*s) were in this region, often near the TATA box or initiator of the associated gene (e.g., *Hsp22* and *Hsp27*). The screen also included more 3′ regions for some (but not all) genes, and detected ten *P* element insertion sites in coding sequence (e.g., for *Hsp70, Hsp22, Hsp68, Hsp83, Hsrω, su(s), act5C,* and *elf;* Figures 2, 4, and 5) or in nearby genes (e.g., nearby *bsg25D* and *CG6296;* Figure 5). Because these regions were surveyed only in those genes in which the UFW primers encompassed them, *P* elements in these regions were excluded from the following comparative analyses.

The data support three of our a priori expectations: (1) that novel TE insertions into *Hsp70* genes should be readily discoverable (Figure 2), (2) that TE insertions should be numerous in *Hsp* genes other than *Hsp70* (Figure 3), and (3) that TE insertions should be rare in genes dissimilar to *Hsp* genes (Figure 3). A total of 29 natural populations (with at least 44 distinct insertions) harbored *P* element insertions into at least one of the five *Hsp70* gene copies (Figure 2), with 13 into the *Hsp70Aa* gene, four into *Hsp70Ab,* and the balance not localized to a specific *Hsp70* gene. *Hsp70* genes also harbored all of the non–*P* transposable elements detected by the screen (one *Jockey* and five *Gypsy*s). *P* element insertions were also numerous (in 30 natural populations with 37 distinct insertions) for *Hsrω* (Figure 4), a single-copy sequence encoding a heat-inducible mRNA. Other heat-shock genes with insertions in more than five natural populations include *Hsp22, Hsp23, Hsp26, Hsp27, Hsp68,* and *Hsp83* (Figure 3).

Excluding *Hsp70,* which presents an expanded target due to its multiple copies, one or more *P* elements were present in the proximal promoter of the first gene set (Gene Set I) in 88 samples (9.8%, 50 populations × 18 genes = 900 samples). In fact, 94% (152) of all 161 *P* insertions discovered in proximal promoters were located in heat-shock genes (*Hsp70* and Gene Set I; Figure 1, inset; Figure 4). By contrast, in Gene Set III, selected for dissimilarity to *Hsp* promoters (see Introduction and Table S1), only 0.2% of the samples (50 populations × 18 genes = 900 samples) included a transposon (one insertion in one population each for CG6295 and CG14030; Figure 5). These transposons, furthermore, seem not to have inserted into the proximal promoter of their genes, but may have inserted into neighboring genes.

The data do not support our last expectation, however, that TE insertions should be common in proximal promoter regions of non-*Hsp* genes similar to those of *Hsp* genes (Gene Set II). Only 0.8% of samples (50 populations × 18 genes = 900 samples) included a TE.

**Figure 6:**
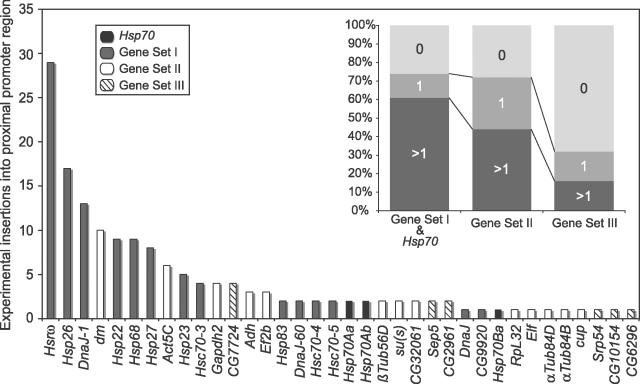
Frequencies of Experimental *P* Element Insertions Reported by FlyBase Database into the Proximal Promoter Regions of Each of the Genes Screened in Natural Populations in the Present Study Data are plotted as in Figure 3. Note that insertions in the different *Hsp70* copies are not combined as in Figure 3. The FlyBase database (http://flybase.bio.indiana.edu/transposons/) terms all tallied *P* element insertions as “transgene insertions.”

Although ordinarily such “natural experiments” do not permit replication, the FlyBase Database (http://flybase.bio.indiana.edu/) records anthropogenic insertions of natural and synthetic transposons. For example, the Berkeley *Drosophila* Gene Disruption Project (BDGDP) has undertaken genome-wide *P* element mutagenesis of laboratory stocks. Indeed, analysis of these data for the three gene sets (Gene Sets I–III) recapitulates the outcome of natural mutagenesis (Figure 3). As of 2004, 157 experimental transpositions into the same genes that we screened in the natural populations are on record, of which 69% were into Gene Set I (*Hsp* genes), 24% were into Gene Set II (*Hsp*-like non-*Hsp* genes), and 7% were into Gene Set III (negative control set with *Hsp*-dissimilar genes; Figure 6). Thus most genes from Gene Set I (14 out of 18; 78%) and II (13 out of 18, 72%) had at least one insertion, whereas only six out of 18 (33%) of Gene Set III had insertions (Figure 6). As in natural mutagenesis, *Hsrω* is distinctive, receiving more than twice as many *P* element insertions as any other gene in the three sets. The two datasets are highly concordant when all genes surveyed are ranked according to number of transposon insertions in the proximal promoter for (1) the 48 natural populations and (2) the synthetic transposon mutant strains (Spearman rank correlation test; *p* < 0.001). In many cases this similarity extends to the specific insertion sites themselves (Figures 4 and 5).

**Figure 7:**
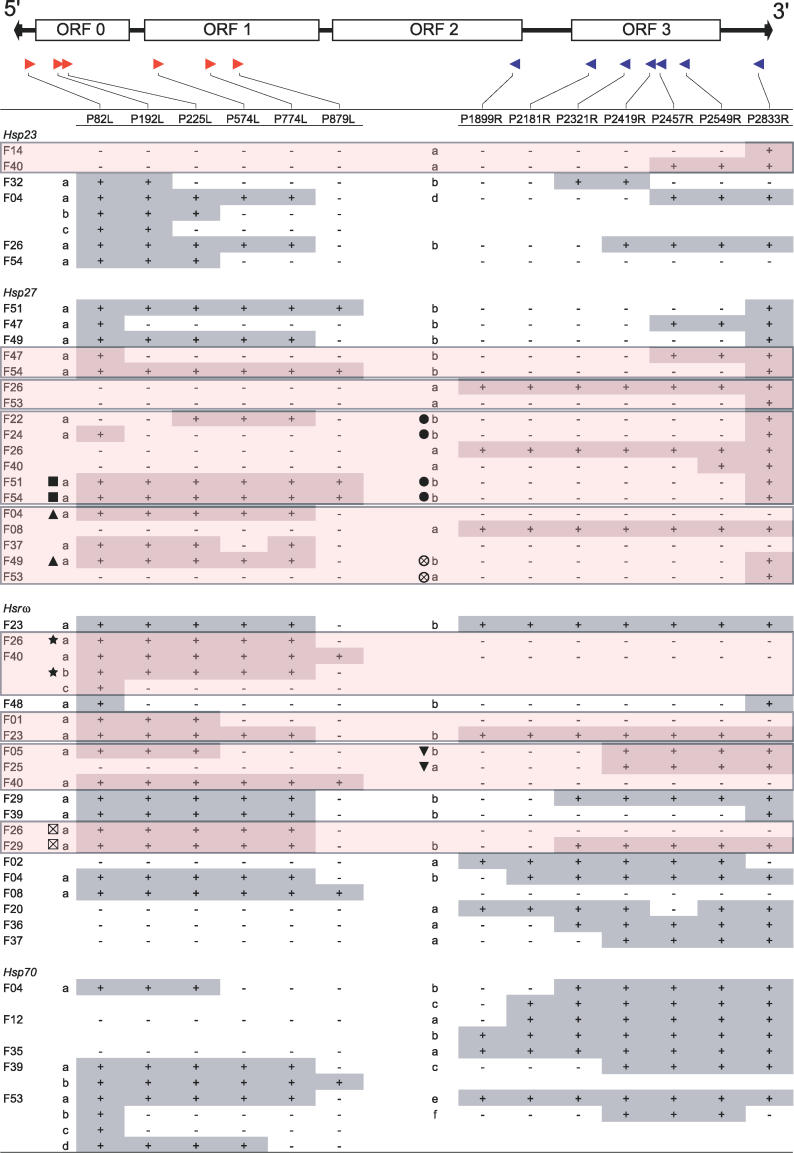
Distinctive *P* Elements Revealed by Re-screening a Random Sample of *P* Element Insertion Sites in Natural Populations for Four Genes, *Hsp23, Hsp27, Hsrω,* and *Hsp70* The *P* element insertion sites were selected from Gene Set I. A plus sign (+) indicates successful PCR amplification with one primer complementary to the focal gene and another complementary to a unique sequence in the *P* element (top), and thus reports the size and orientation of the *P* element; a minus sign (−) indicates no amplification. Table S7 provides sequences of these primers. At each insertion site in a population, one to six distinctive *P* elements segregated; these are designated a–f. For *Hsp23, Hsp27,* and *Hsrω,* nine insertion sites shared by two or more natural populations (indicated by boxes) and 17 unique insertion sites were re-screened. Amplicons that share a symbol (filled square [█], filled triangle [▴], filled circle •], etc.) occurred at the same integration site in different populations and were indistinguishable by size or orientation. For *Hsp70,* a five-copy gene in natural populations [17], the specific gene of insertion was not determined; thus, each distinctive amplicon (a–f) could represent insertion(s) at the same site in one to five of the *Hsp70* genes. For population codes see Figure 1. ORF, open reading frame.

According to the Fisher exact test, the natural *P* element insertions were more numerous in Gene Set I than in Gene Set II (*p* = 0.018) and in Gene Set III (*p* = 0.002). By contrast, experimental *P* element insertion in Gene Sets I and II were not significantly different (*p* = 1.0), but more numerous in each than in Gene Set III (*p* = 0.004 and *p* = 0.010, respectively).

### Characteristics of *P* Elements within Promoters

The insertion sites of *P* elements in the *Hsp* promoters were themselves highly clustered, with up to eight populations putatively receiving different insertions at the same site (Figure 2). The elements also varied in orientation relative to the associated coding sequence, with no apparent orientation preference (Figure 7).

### Re-screening Establishes that *P* Elements Are More Numerous than the UFW Screen Indicates

Of the transposon inserts discovered, 47 were clearly unique (i.e., no two populations shared the same transposon at the same location in the same gene). By contrast, many different populations exhibited identical insertion sites, with up to eight populations showing *P* element insertions at the same position (this, for example, in the proximal promoter of *Hsp70;* Figure 2). As cited in the methodological concerns (above), these could represent insertion(s) of a *P* element into a common ancestral population and its vertical transmission into daughter populations, rather than independent multiple insertion events at the same site, and hence overestimate *P* element insertions. Alternatively, at any insertion site detected by UFW, multiple but similarly sized *P* elements might have inserted and presently segregate in any natural population so that UFW screening might underestimate TE insertions.

To re-examine our above estimate of TE abundance in natural populations, 43 insertion sites (17 unique, with nine sites shared in two to six populations) in three different genes *(Hsp23, Hsp27,* and *Hsrω)* were chosen for re-analysis. We used a PCR-based technique that reports both the size and orientation of the *P* element (Figure 7).

In the 17 unique sites are 30 *P* elements distinguishable by size and orientation. In the nine shared sites are 30 *P* elements distinguishable by size and orientation. Of these, five are present in two populations, and one is present in four populations (Figure 7). In all but one of these instances, the shared *P* element is singular in a first population and one of two to three forms segregating in a second population. In the remaining case, in *Hsp27* in populations F51 and F54, two *P* elements segregate at the same insertion site in both populations. A similar re-screening of the *Hsp70* genes at a “single” insertion site in each of five populations (Figure 7) detected one to six distinctive *P* element variants at each site (15 total). Each was localized relative to a sequence shared by the five *Hsp70* genes in natural populations, but not to a specific *Hsp70*. Thus, each *P* element could be present at the same insertion site in one to five *Hsp70* copies, with the tally of 15 under-representing the actual number of insertions.

In summary, excluding the re-screened *P* elements in *Hsp70,* 60 distinctive *P* element variants were found at the 43 sites re-analyzed, suggesting that the UFW screen undercounted distinctive *P* elements by nearly 30%. Corrected for this undercount, all 117 *P* element insertion sites in the three gene sets likely harbor 163 distinctive transposable elements in the natural populations, or 225 with those in the *Hsp70*s included.

These distinctive *P* elements may represent distinctive insertion events, distinctive evolution after common insertion events, or both. To estimate a lower bound for the frequency of insertion events, distinctive *P* elements at the same 43 insertion sites were re-tallied based on orientation only (and not size). Any two *P* elements with opposite orientation likely arose from independent insertion events rather than from evolution after a common insertion (but more than two independent insertions cannot be distinguished). On this basis, 43 distinctive *P* elements are distinguishable at the 43 sites. In other words, the *P* element tally based on UFW likely does not overestimate the number of independent insertion events, although it may underestimate this number.

### Allelic Frequencies of the *P* Element Insertions

Individual *P* elements varied in both nucleotide sequence and allelic frequencies in populations. A prior study [14] suggested that transposition into the proximal promoters of *Hsp* genes can be selectively advantageous because of its impact on *Hsp* gene expression. Moreover, although deleterious TEs might be inactivated if not purged from populations [32,33], adaptive TEs might be maintained at high frequencies or modified. Surveys of several randomly selected populations (Table 2; Figure 7) are consistent with the simultaneous modification and maintenance of TEs. All *P* elements discovered whose size was determined were less than full length. The allelic frequencies of *P* elements at each site surveyed (Table 2) varied considerably among populations, ranging from very low (e.g., in F40 for *Hsp22*) to high (e.g., in F04 for *Hsp27*) or even fixation (e.g., in F51 for *Hsrω*). Populations also differed in the number of different variants of the *P* element inserted in a particular region of a gene or the number of insertion sites (Table 2), and frequency and number of insertion sites are not correlated. Population F40, for example, harbors three different *P* elements at two different sites in *Hsrω*. The allelic frequencies, however, of these insertions are low (6%), whereas that for a single *P* element in *Hsp27* in population F04 is much higher (85%; Table 2; Figure 4).

**Table 2:**
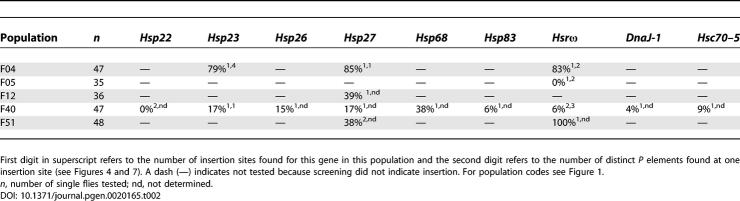
Frequency of *P* Element Insertions in the Promoter Region of Nine Heat-Shock Genes in Different Populations

In six (12%) natural populations (F09, F10, F11, F16, F41, and F42; Figure 1), no *P* elements had inserted in the proximal promoter regions of the genes under investigation according to UFW. Re-screening these populations with PCR revealed no *P* elements; *P* elements either are absent in these populations or too distant from one another to support PCR amplification. Interestingly, all six populations are geographically adjacent (Figure 1). Only about half (ten out of 21) of the African populations harbored two or more *P* element insertions, in contrast to 78% of the populations outside Africa. The population with the most numerous insertions (*n* = 14, F40 from Marrakech), however, is African. Although specific mobile element insertions vary in frequency along geoclimatic gradients [14–17,21], in our data, the number of insertions bears no apparent relationship to latitude or climate.

## Discussion

We have hypothesized that heat-shock genes as a class are distinctively evolvable because TEs integrate into their proximal promoter regions at unusually high rates, creating unique regulatory variation on which evolutionary processes such as natural selection can act. Here we test a major component of this hypothesis, that heat-shock proximal promoter regions are especially susceptible to the integration of TEs. TEs and other repetitive elements constitute more than 20% of the D. melanogaster genome [22], comprising 6,013 specific elements in more than 120 families [23,26]. Although these numbers are less than for other multicellular eukaryotes (e.g., ~45% in humans) [34], they nonetheless establish that TEs are numerous and diverse in D. melanogaster. TEs in *Drosophila,* moreover, are active, accounting for more than 50% of spontaneous mutation [35] (versus 0.2% in humans [36]). Accordingly, the discovery of more than 170 additional TEs in natural *Drosophila* populations is, in itself, unremarkable. What is striking is the predilection of these TEs for insertion in the proximal promoters of one class of genes, their persistence after insertion, and the fact that almost all are *P* elements.

### Methodological Issues

Deducing this susceptibility from compilations of insertion sites is prone to bias unless (1) all TEs are detected, and (2) similarly sized regions are compared. With respect to (1), UFW is inclusive of all insertions, even currently undescribed TEs, because it is sensitive to size and not sequence [28]. UFW can be problematic, however, if deletion exactly counterbalances the insertion of novel elements and/or if its PCR steps favor amplification of small products. With respect to (2), we scrutinized equally sized regions of the three gene sets.

One prospective weakness of this approach is that no two selected genes are the same and thus may differ as targets in ways not relevant to the main hypothesis. For example, TEs might be rare in those sets with many genes in regions of high recombination, which are thought to disfavor the persistence of TEs [37]. Genes that are located in regions of high recombination [38] are equally numerous in the three gene sets (Table 3; Fisher exact test, *p* = 0.7). In fact, those genes in Gene Set I most numerous in *P* element insertions are in highly recombining region in most cases. At any rate, to guard against other unforeseen confounding factors, we surveyed 18 genes in each set on the assumption that similarities would manifest themselves if robust. Another prospective weakness is that any given natural population may be unrepresentative of entire species. To compensate, we surveyed 48 populations (and a reference strain). Thus, although no screen is free of biases, the results provide a reasonably unbiased assessment of naturally occurring TE insertions in the proximal promoters of three contrasting sets of genes.

**Table 3:**
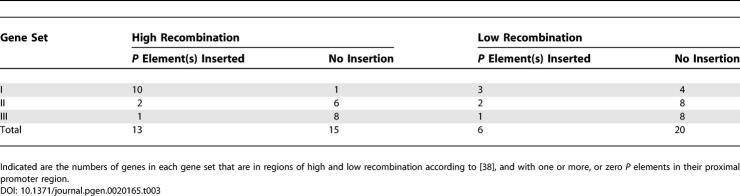
*P* Element Insertions into Genes in Regions of High and Low Recombination

### Are Heat-Shock Promoters Conducive to *P* Element Insertion, and Why?

The abundance of *P* elements in *Drosophila* heat-shock promoters may arise from the species' distinctive (but common) ecological niche. Like many organisms, D. melanogaster often undergoes hyperthermia in nature (e.g., [39,40]). *Drosophila* infests necrotic fruit, wherein eggs, larvae, and pupae are prone to heat stress. At the biochemical level, this hyperthermia is deleterious to proteins and membranes, and for the former may initiate a cytotoxic cascade of denaturation and aggregation of proteins [41]. At the organismal level, this heat stress compromises development, reproduction, and survival. A primary and important response to heat stress is therefore the expression of heat-inducible molecular chaperones, which can deter protein aggregation, target damaged proteins for degradation, help non-native proteins refold in the cell, and/or remove proteins from aggregates for refolding or degradation [42]. Given these essential roles, heat-inducible molecular chaperones are poised for rapid and massive accumulation upon heat shock [41]—hence their original name, “heat-shock protein.” Indeed, several distinctive features of the heat-shock genes of complex eukaryotes appear to facilitate heat-induced expression: constitutively decondensed chromatin, nucleosomes positioned outside the proximal promoter, a pre-assembled (but paused) polymerase apparatus, a pre-expressed, inactive, but readily activatable transcription factor (HSF), and the absence of introns needing splicing (e.g., [12,43–48]). Each feature can be viewed as an elimination or minimization of a time-consuming step in gene expression, and is thus an appropriate component of an emergency response to rapid and unpredictable thermal damage.

As explained in the Introduction, these same features give TEs accessibility to chromatin, which could facilitate insertion [2]. Because *Hsp* genes share some of these features and represent extreme manifestations of others, Lerman et al. [15] suggested that proximal promoters of *Hsp* genes in general were natural “hotspots” for TE integration. Although this suggestion was consistent with the discovery of naturally occurring TEs in *Hsp70* genes' promoters [15], (1) these TEs were few, (2) with few exceptions [49], naturally occurring TEs had not been discovered in other *Hsp* genes, and (3) TEs were not known to be comparatively rare in the proximal promoter regions of non–heat-shock genes. Three independent lines of evidence now establish that insertions of one TE, *P* elements, are common, not only in *Hsp70* promoters, but also in other (single copy) heat-shock promoters:

The present screen of natural populations documents (1) numerous *P* elements inserted into the proximal promoters of *Hsp70* genes (Figure 2), (2) numerous *P* elements inserted into the proximal promoters of *Hsp* genes other than *Hsp70* (Figure 3), and (3) few or no *P* elements inserted in the proximal promoters of *Hsp*-dissimilar genes (Figure 4). That *P* elements have inserted in non-transcribed sequence is not unexpected [50], but our comparison is of identical regions in each gene set. If transposition into a region is solely a function of that region's representation in the euchromatin, then each gene set should accumulate equal numbers of *P* elements. The UFW and *P* element screens, by contrast, detect 152 (94.4%) in the heat-shock genes, seven (4.4%) in Gene Set II, and only two (1.2%) in Gene Set III (Figure 1, inset; Figure 3). As explained, the two in Gene Set III actually reside in nearby genes (Figure 5) and could therefore be excluded (although we have not done so).

The accompanying survey of transgene insertion experiments (Figure 6), mainly from a genomic mutagenesis scheme that relied on *P* elements [8], has largely the same outcome (in number and position), at least for heat-shock genes (*Hsp70* and Gene Set I) and the negative gene set (Gene Set III).

Shilova et al. [10] mobilized *P* transposon constructs *(EPgy2)* adjacent to the *Hsp70A* gene cluster and documented numerous new insertions into *Hsp70* via local transposition (Figure 2C). They recovered 46 independent insertions of which, remarkably, 50% were into the same two nucleotides, −96 and −97, which is in correspondence with our results (Figure 2C). These two nucleotides harbor 12 (27%) of the 44 *P* elements we discovered in *Hsp70* genes in natural populations, and represent a remarkable conjunction of DNase I hypersensitivity, adjacency to GAGA factor binding sites, absence of nucleosomes, and insertion site preference [10].

This largest number of *P* element insertions, both natural and transgenic, is into *Hsrω,* a highly conserved single-copy gene that encodes a heat-inducible mRNA, but no protein [51]. Like many non-coding RNAs, *Hsrω* transcripts play diverse regulatory roles [51], including regulation of distribution of RNA binding proteins [52] and the suppression of non-*Hsp* gene expression upon heat shock [53], and suppression of polyglutamine neurotoxicity [54]. In 30 (62.5%) of the 48 natural populations surveyed, a *P* element has integrated in *Hsrω,* with 70% distinctive integration events into nucleotides −89 to −161 upstream of the transcription start site. Why this region is so susceptible to *P* element integration is not apparent, but these insertions are not into any major regulatory element [55], and most of this region is not conserved within D. melanogaster.

Two additional aspects favoring successful *P* element integration in the germline are expression of the host gene, and the occurrence of this expression in germ cells prior to meiosis, an embryonic process in *Drosophila* [9,56,57]. Gene Set III, essentially negative controls, was selected for restriction to narrow developmental windows and low levels of expression (Table S1). Genes in this set also exhibit neither constitutive chromatin decondensation, nor positioned nucleosomes, nor regulation via polymerase pausing; i.e., none of the attributes hypothesized to favor TE integration in *Hsp* promoters. Accordingly, the near absence of TEs discovered in this set and their complete absence from the proximal-promoter region is consistent with the above conclusions.

The paucity of transposons discovered in Gene Set II, the “*Hsp*-like” genes, is more difficult to reconcile, however. One possible explanation is that the “true” *Hsp*s are prone to massive environmentally induced expression during the embryonic period in which germline transformation is possible, whereas the *Hsp*-like genes are not. As we discuss below, an alternative, but non-exclusive, explanation is that natural selection more effectively eliminates TEs from *Hsp*-like genes than from *Hsp* genes.

### Why Have *P* Elements Persisted in the Promoters of *Hsp* Genes in Nature?

A common view is that TE insertions into genes are generally deleterious because mutations in general are usually deleterious and are therefore eliminated from the genome by purifying selection. Evidence includes the deleterious phenotypes that commonly result when TEs integrate into genes (for *P* elements, reviewed by [58]), the comparative rarity of TE insertions into coding or regulatory regions of genes, the low frequencies of intragenic TEs segregating in natural populations [18,19], and the rapid evolution of suppression of transposition in natural populations (e.g., *trans*-acting RNAs, DNA methylation, and specific antisense RNA). For *Drosophila,* instances in which intragenic TEs are beneficial are so rare that accounts of such instances are newsworthy [59–62]. Why, then, are *P* elements in the *Hsp* promoters of naturally occurring *Drosophila* so numerous?

One explanation is that *P* elements are distinctive transposable elements in several respects. First, unlike other TEs (for example, the *Ty* elements of *Saccharomyces,* which insert in specific sites of specific genes [2]), the information content of *P* insertion sites is relatively low [63]. *P* element insertions thus are general and robust reporters of exposed chromatin. Second, *P* elements have invaded the D. melanogaster genome relatively recently. Biogeographic patterns of *P* element occurrence in D. melanogaster and the recent near-disappearance of D. melanogaster populations without *P* elements together suggest that *P* elements invaded the D. melanogaster genome within the last century [29–31]. Since then, *P* elements spread widely and effectively throughout the D. melanogaster genome, likely due to self-regulation and hybrid dysgenesis [29,64].

TEs typically undergo excision or degeneration with time. Indeed, whereas full-length *P* elements encode a transposase and are thus autonomous, every *P* element discovered in the UFW screen whose size we have determined is less than full length. Thus, the occurrence of *P* elements in the *Hsp* proximal promoters could be transient, representing a successful genomic invasion that has not yet been purged. Although this may be so, populations we have surveyed average two to three *P* elements in their *Hsp* promoters. Given that natural populations typically carry no more than 50 *P* elements distributed throughout their more than 13,000 genes [65], their abundance in *Hsp* promoters is remarkably high.

Importantly, in natural *Drosophila* populations TEs in *Hsp* promoters may be advantageous and therefore persist via positive selection (e.g., [21]). Although *Hsp*s typically encode proteins that can function as molecular chaperones, these proteins have numerous other functions including intra- and extra-cellular signaling (small *Hsp*s, *Hsp60,* and *Hsp70*) [66], regulation of cell cycle and apoptosis [67,68], and maturation and regulation of nuclear receptors, among others [66,69]. *Hsp90* (encoded by *Hsp83* in *Drosophila*) alone interacts with numerous (>200) client proteins in the cell [70]. Thus, whereas massive *Hsp* accumulation often may be advantageous for temperature tolerance and mitigation of thermal damage, in the absence of heat stress, stringent regulation of *Hsp* levels may be essential. Indeed, unbridled expression of *Hsp* genes in the absence of heat stress is often deleterious (reviewed by [15]). The typical phenotype of TE insertions into *Hsp70* promoters is decreased gene expression [14], and natural *P* insertions have similar phenotype in *Hsp26* in population F32 (unpublished data). Accordingly, selection may favor the retention of TEs in *Hsp* promoters because these elements reduce expression of a situationally harmful protein. Natural and experimental selection can either increase or decrease *Hsp* expression depending on which outcome a given thermal environment favors (reviewed by [15]). *P* element insertion may thus simply be an opportunistic way to suppress *Hsp* activity in the wild.

Intragenic TEs segregating in natural *Drosophila* populations are typically at very low frequencies, consistent with their usually deleterious phenotypes (see above). On the other hand, intragenic TEs considered advantageous, by contrast, are typically at much higher frequencies, if not fixed, in natural populations [13,20,71]. In our survey, although frequencies of *P* elements at specific sites varied considerably in natural populations, these sometimes were much higher than those invoked as evidence for deleterious or neutral phenotypes [18,19]. Ten of the 16 *P* element insertions we arbitrarily chose for examination in detail were at population frequencies ≥15%, seven at ≥35%, and four at ≥75% (Table 2). This outcome resembles that for prior studies of TEs in *Hsp70* promoters in natural populations, in which frequencies were high and varied inversely with *Hsp70* protein levels [14–17,21]. A testable prediction is that experimental evolution in contrasting thermal regimes should be capable of altering *P* element frequencies in *Hsp* genes according to the phenotypes of *P* element insertions; i.e., increasing *P* element allelic frequency when beneficial and decreasing frequency when deleterious. The same prediction ought to be applicable to other instances in which TE-derived sequences modify a host function and have been assimilated by the host genome [1,20,49,72,73].

Interestingly, in the transgene lines, laboratory strains that are intentionally isolated from natural selection, the *Hsp* and *Hsp*-like genes included in our survey received transposon insertions at similar frequencies (Figure 6, inset) and positions. In nature, by contrast, the proximal promoters of the *Hsp*-like genes (Gene Set II) were relatively depauperate *P* elements. This contrast further implicates positive or balancing selection for maintenance of *P* elements in the *Hsp* genes (Gene Set I and *Hsp70*). In other words, in nature, selection may routinely purge *P* elements from the proximal promoters of non-*Hsp* genes and/or favor insertions in *Hsp* genes. It suggests that accessibility of chromatin to *P* element integration may be necessary, but not sufficient, to generate stable *P* element insertions in the proximal promoters of these genes in nature because of negative or purifying selection.

Heat-shock genes are a component of an ancient, but effective, response to acute thermal stress in the natural environment, and include features that facilitate rapid and massive gene expression (e.g., [40,42,74]). These same features, however, may facilitate the integration of *P* elements into *Hsp* promoters, which in turn affect gene expression. These intragenic *P* elements thereafter may segregate in natural populations and are a form of genetic variation upon which natural selection and other evolutionary processes may act. The present study and another (unpublished data) establish that this scenario is not specific for a single multicopy gene *(Hsp70)* in a small number of natural populations as previously described, but generally applicable to *Drosophila* populations worldwide and to the entire heat-shock genome. From this perspective, intragenic TEs in natural *Drosophila* populations are less newsworthy exceptions [59–62] than a normal expectation. The near-exclusive involvement of *P* elements and the frequencies of these elements establish, moreover, that the TEs in *Hsp* promoters are not the remnant of an ancient event, but a manifestation of active and ongoing microevolution in natural populations.

Heat-shock genes have been posited to make a special contribution to evolvability [75]. The capacity of their products—molecular chaperones—for conformation-specific recognition of diverse client proteins has led to their involvement in diverse regulatory processes and their assignment to diverse structural roles. Importantly, molecular chaperones may transiently suppress conformational mutations in client proteins, thereby protecting such variation from selection under routine conditions, but exposing it during stress [76]. TEs have likewise been posited to make a special contribution to evolvability [1]. They are the most widespread and effective of natural mutagens, can redistribute genetic material throughout the genome, and form the recombinational substrate for much gene duplication, retroposition, and creation of hybrid genes. Here we show that these two components of evolvability intersect: heat-shock genes as a class are distinctively prone to the integration of at least one TE, the *P* element, and that, accordingly, the expression of heat-shock genes may evolve distinctively from the rest of the genome.

## Materials and Methods

### *Drosophila* strains.

The *Drosophila* stocks used in this survey were derived from wild-caught flies and were maintained as isofemale lines with the exception of F32, F51, and F52. Flies were from 51 different worldwide locations (Figure 1), plus a reference strain. Most lines were obtained from Dr. Jean David (Centre Nationale de la Recherche Scientifique, France), and have been the subject of previous investigations [77–80]. In addition, strains F01 (14021-0231.21), F02 (14021-0231.22), F03 (14021-0231.23), F04 (14021-0231.25), and F05 (14021-0231.26) were obtained from the *Drosophila* Species Stock Center, Tucson, Arizona, whose reference numbers are in parentheses. The reference strain (F06: *y* ^1^ *; cn* ^1^ *bw* ^1^ *sp* ^1^), “the Celera strain,” was the strain whose genome has been sequenced [25]. This strain is free of *P* elements [26,80]. Strain F32 was provided by Dr. Michael Rose (University of California, Irvine) and is one of the “base” or control strains used in his studies of laboratory evolution [81]. Strains F51 and F52 were from the north- and south-facing slopes, respectively, of “Evolution Canyon” (Lower Nahal Oren, Israel) [82]. Additional strains and origins include: F49, Dr. Arne Mooers (Simon Fraser University, Canada); F48 and F50, Jennifer Shirriffs (La Trobe University, Australia); and F53 and F54, Dr. Masayoshi Watada (Ehime University, Japan) [83,84]. All live flies were reared on a yeast, cornmeal, molasses, and agar medium at 25 °C.

### DNA isolation.

Bulk samples of genomic DNA were extracted from 2 × 50 individual adults for each population. Flies were fresh or preserved in 70% or 100% ethanol. Ethanol-preserved files were air-dried and washed in 500-μl phosphate-buffered saline solution (PBS) for 2 min prior to DNA isolation. The washing buffer was removed and another 180-μl PBS was added for grinding. Total DNA was extracted according to [28].

### Gene sets.

Genes (Tables 1 and S1) were selected and grouped into sets for analysis a priori according to the following criteria: (1) the nearly identical *Hsp70* genes [17]. These were included for re-analysis because of the prior discovery of several TEs in their 5′-flanking regions (see Introduction). These genes are arranged in two clusters (*Hsp70A* at 87A7, comprising *Hsp70Aa* and *Hsp70Ab,* and *Hsp70B* at 87C1, comprising *Hsp70Ba, Hsp70Bb, Hsp70Bbb* (if present), and *Hsp70Bc*); (2) other heat-shock genes (Gene Set I) [74,85]. Although all genes included in this group of genes increase in expression upon heat shock or other proteotoxic stresses, most (unlike *Hsp70*) [86] are expressed constitutively. Also, although several subsets of these genes share similarities in sequence, they are not multicopy genes in the same sense as the *Hsp70*s, which encode proteins of identical sequence [17]; (3) genes resembling heat-shock genes in regulation of expression, chromatin configuration, associated promoter elements, etc. (see Introduction) (Gene Set II). These were identified from literature reports (Table S1); and (4) Genes dissimilar to heat-shock genes or with no known features similar to those of heat-shock genes (Gene Set III). These were initially selected from the data of Arbeitman et al. [87], available online (http://flygenome.yale.edu), according to least expression throughout the *Drosophila* life cycle. All else equal, genes with limited embryonic expression were preferred. Initially selected genes were discarded if a literature search disclosed characteristics that might qualify them for inclusion in Gene Set II. In most cases, genes included in this set had not been studied in detail when the set was compiled, or had hypothetical functions according to sequence homology with better-studied genes (Table S1).

### Transposable element screening.

We executed two screens with differing characteristics. The first, a UFW screen reported size polymorphism in amplicons between the reference strain Celera (F06) and natural populations. We modified UFW as originally described [27] to minimize false positives, and included an additional nested PCR step [28]. A total of 5 μl of genomic DNA (100 ng) extracted in bulk from 2 × 50 adult flies was used for each reaction. Vista (http://genome.lbl.gov/vista/index.shtml) and Primer 3 (http://frodo.wi.mit.edu/cgi-bin/primer3/primer3_www.cgi) were used to design six to seven UFW primers for all 55 genes binding either to a highly conserved region in the CDS (coding sequence) of the gene or to a neighboring gene (Tables S2, S3, and S4). Because of the extraordinary conservation of *Hsp70* coding sequence [17], UFW primers specific to each of the five to six *Hsp70* genes were impossible to design. Therefore the UFW screen of *Hsp70* indicated amplicon size polymorphism in one or more of the genes.

In brief, UFW is a non-restrictional, non-ligational genome walking method that uses primer complementary to a known sequence (in our case, coding sequence), random primers, PCR, and nuclease digestion in combination to amplify unknown sequence flanking the known sequence. Insertions (e.g., TEs) or deletions present in the amplified unknown region are detectable because they increase or decrease the size of the amplicon. TEs were subsequently identified by sequencing and localized via the PCR screen described below. More detailed descriptions of the assay, guidelines for primer design, and exemplary gel images are in Myrick and Gelbart [27] or Walser et al. [28].

About 15% of all UFW reactions initially failed or produced only modest amplification even after the nested PCR step, and were therefore repeated with a different set of primers. Populations F18, F50, and F52 were removed from the dataset after the reaction failed for multiple genes and primer sets. Presumptive positives (defined as extra bands in the UFW footprint compared to the reference strain) were cloned and then sequenced for identification (at least one per gene). Topo TA Cloning Kit (Invitrogen, Carlsbad, California, United States) for amplicons fewer than two kilobases and Topo XL PCR Cloning Kit (Invitrogen) for larger fragments were used. FlyBase query and Repeat Masker software (with the latest release of the RepBase database update; http://www.girinst.org) were used to assess the similarity of insertions with known sequence.

When the vast majority of insertions (98%) that UFW screening revealed were *P* elements, a second PCR-based screen was designed to confirm the UFW results and discover further insertions. In this second screen, one primer site was chosen in the conserved region of the associated gene, and the other in the 31-bp (base pair) inverted terminal repeat region of the *P* element. The *P*-specific PCR screen also included a further population from Israel (F51) and two populations from Japan (F53 and F54). Although primers for the second screen were gene specific, all positive samples (samples amplified in the second screen) were re-screened with another primer specific to the conserved region of the associated gene. PCR reactions with only one primer, the *P* element–specific primer, served as a control for inadvertent amplification of sequence between multiple *P* element insertions. Additionally, we sequenced 75% of all positive samples, including all fragments bigger then 450 nt. For amplicons smaller then 450 nt, a −6FAM–labeled *P* element–specific primer was used, and the PCR products were sized on an ABI 3730 DNA sequencer with LIZ-500 as internal size standard using Genemapper 3.0 (Applied Biosystems, Foster City, California, United States).

### Control screening.

To confirm that the discovery of *P* elements was not due to some inadvertent bias of the UFW screen, we performed additional PCR-based screens for six TEs common in the Celera strain (*roo, 1360, 297, Jockey-*family, *I* elements, and *Gypsy*-family elements) [26] and still active in the genome. The same PCR-based approach as for *P* element screening was used, with one primer specific for a conserved region of the associated gene and the other for the TE being screened. Because these TEs lack inverted terminal repeats, two element-specific primers were used to establish TE orientation. TE-specific primers were designed from alignments of TE sequences deposited in the National Center for Biotechnology Information (NCBI) GenBank database (Table S5). For all populations, the promoter region of *Hsp70* was screened for these six TEs. Furthermore, two genes from each set of genes were likewise re-screened for four populations (F04, F40, F53, and F54).

### Screening for deletions.

A final PCR-based screen used primers (Table S6) complementary to conserved regions in a gene of interest and a neighboring gene, and thus amplified the entire intergenic/intervening region. Because PCR preferentially amplifies smaller sequences in size polymorphisms, this screen was expected to detect primarily deletions and small insertions rather than larger TEs at a low frequency.

### Frequency of insertion.

For selected genes and five populations (F04, F05, F12, F40, and F51) the frequency of *P* element insertions was estimated by analyzing 35–48 individual flies per population (Figure 1). DNA was purified from single flies according to the manufacturer's recommendations (Puregene DNA Purification Kit; Gentra System, Minneapolis, Minnesota, United States).

### Distinguishing *P* element insertion events.

*P* elements discovered at the same site in multiple populations might have been inherited from a single ancestral population or have inserted independently. Also, any *P* element discovered at a site might either be singular or represent multiple elements segregating in a population. To distinguish among these alternatives, 43 *P* element insertions (at different sites with populations sharing zero to six *P* transposons at each site) from three different genes *(Hsp23, Hsp27,* and *Hsrω)* were re-screened with a PCR-based technique that reports both the size and orientation of the *P* element (Figure 7; Table S7). This screen exploited the tendency of the internal regions of *P* elements (but not their termini) to truncate during evolution [3], but in highly variable fashion. From an alignment of full-length and truncated *P* element sequences deposited in the NCBI GenBank database, six forward and seven reverse primer sites were chosen. The corresponding amplicons (indicated by “+” in Figure 7) are indicative of the size and orientation of the corresponding *P* element.

### Transgenic insertion sites.

The flanking region of all experimental *P* element transpositions thus far reported by the FlyBase Database (http://flybase.bio.indiana.edu) were used to characterize insertion site for the genes of the different gene sets (Gene Sets I–III). In addition, we also included locations of *EPgy2* element insertions for *Hsp70* recently described by Shilova et al. [10] for comparison with the naturally occurring *P* elements. Spearman rank correlation was used to test for a monotonic relationship between the natural and the transgene insertion sites for the different genes. The Fisher exact test for count data was used to compare the number of gene with element inserts in the three gene sets.

### Other polymorphisms.

The UFW screen also detected several insertions/deletions, presumably deletions by parsimony (Figure 4). In population F40, a 1,381-nt deletion is in the 5′ region of *Hsp27*. The deletion occurs upstream of the TATA box and removes all five heat-shock elements (HSE). Another deletion of 565 nt occurs in *Hsp68* in population F21. This removes all four HSEs, the TATA box, and the initiator. Population F01 exhibited a pair of deletions in *Hsrω,* in which 17 nt and 5 nt of the 5′ and 3′ regions flanking the TATA box were absent. Screens of individual *Drosophila* (*n* = 50) suggest that these deletions segregate in populations at very low frequencies (<2%).

## Supporting Information

Table S1 Supplementary Information on Genes in the Three Gene Sets (219 KB PDF)

Table S2 UFW Primers for Gene Set I (36 KB PDF)

Table S3 UFW Primers for Gene Set II (44 KB PDF)

Table S4 UFW Primers for Gene Set III (31 KB PDF)

Table S5 Transposable Element–Specific Primers for Additional Screens (80 KB PDF)

Table S6 Primers for Intergenic Regions (58 KB PDF)

Table S7 *P* Element–Specific Primers for Orientation and Length Determination (299 KB PDF)

## Abbreviations

TE: transposable element
UFW: universal fast walking
