# Supplementary material for: Heat-Shock Promoters: Targets for Evolution by P Transposable Elements in Drosophila
Source: PLoS Genet. 2006 Oct 6;2(10):e165. doi: 10.1371/journal.pgen.0020165 (PMC1592238; doi:10.1371/journal.pgen.0020165)
Supplement: Supplementary file 1 [file pgen.0020165.st001.pdf]

Table S1: Supplementary information on genes in the three gene sets

Walser et al. 2006
Heat-shock promoters: targets for evolution by *P* transposable elements in *Drosophila*

Sources used for the tables and for further information:

(1) Drosophila developmental gene expression timecourse
http://genome.med.yale.edu/Lifecycle/

Drosophila Developmental Gene Expression Timecourse (http://genome.med.yale.edu/Lifecycle/).

Color coded time course for gene list: 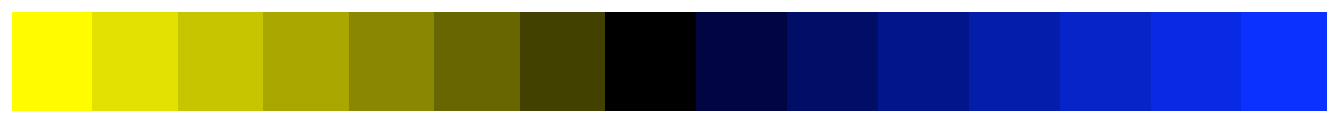

Yellow color represents high relative levels of expression while blue represents low levels. The brightest color is three-fold or greater differential from the reference black

(2) FlyBase - a database of the Drosophila Genome
http://flybase.bio.indiana.edu/

(3) FlyGRID - a database of genetic and physical interactions developed
http://biodata.mshri.on.ca:80/fly_grid/servlet/SearchPage

(4) MitoDrome – a database with the aim to annotate the complete set of *Drosophila melanogaster* nuclear genes encoding for mitochondrial proteins in order to contribute to their functional characterization.
http://www2.ba.itb.cnr.it/MitoDrome/

(5) BDGP Gene Expression Summary
http://www.fruitfly.org/cgi-bin/ex/bquery.pl?qpage=entry&qtype=summary

Gene Set I. Heat-inducible genes, *hsp* genes, chaperone genes. ID: FlyBase ID; SYM This is the standard abbreviation (gene symbol) for the name of the gene.; Location: Chromosome arm and Location of the gene on the cytological (polytene chromosome) map. Developmental Gene Expression Timecourse (for color code see above)

					Metamorphosis		Adult		
ID	SYM	Location	Embryo	Larvae	Prepupae	Pupae			Ref.
CG4460	*Hsp22*	3L-67B2							1-5
CG4463	*Hsp23*	3L-67B3							1,5
CG4183	*Hsp26*	3L-67B2							1,5
CG4466	*Hsp27*	3L-67B3							1,5
CG5436	*Hsp68*	3R-95D11							5
CG18743	*Hsp70Ab*	3R-87A3							1,5,6
CG1242	*Hsp83*	3L-63B11							5
CR31400	*Hrsω*	3R-93D4							7
CG10578	*DnaJ-1*	3L-64E5							8
CG12240	*DnaJ-60*	2R-60C1							9
CG5504	*DnaJ*	2R-59F6							10
CG9920		3R-88A8							
CG12101	*Hsp60*	X-10A4							11
CG2830	*Hsp60b*	2L-21E2							12
CG7756	*Hsc70-2*	3R-87D10							13-15
CG4147	*Hsc70-3*	X-10E3-4							14,15
CG4264	*Hsc70-4*	3R-88E4							13-15
CG8542	*Hsc70-5*	2R-50E6							14,15
CG4167	*Hsp67Ba*	3L-67B21							5

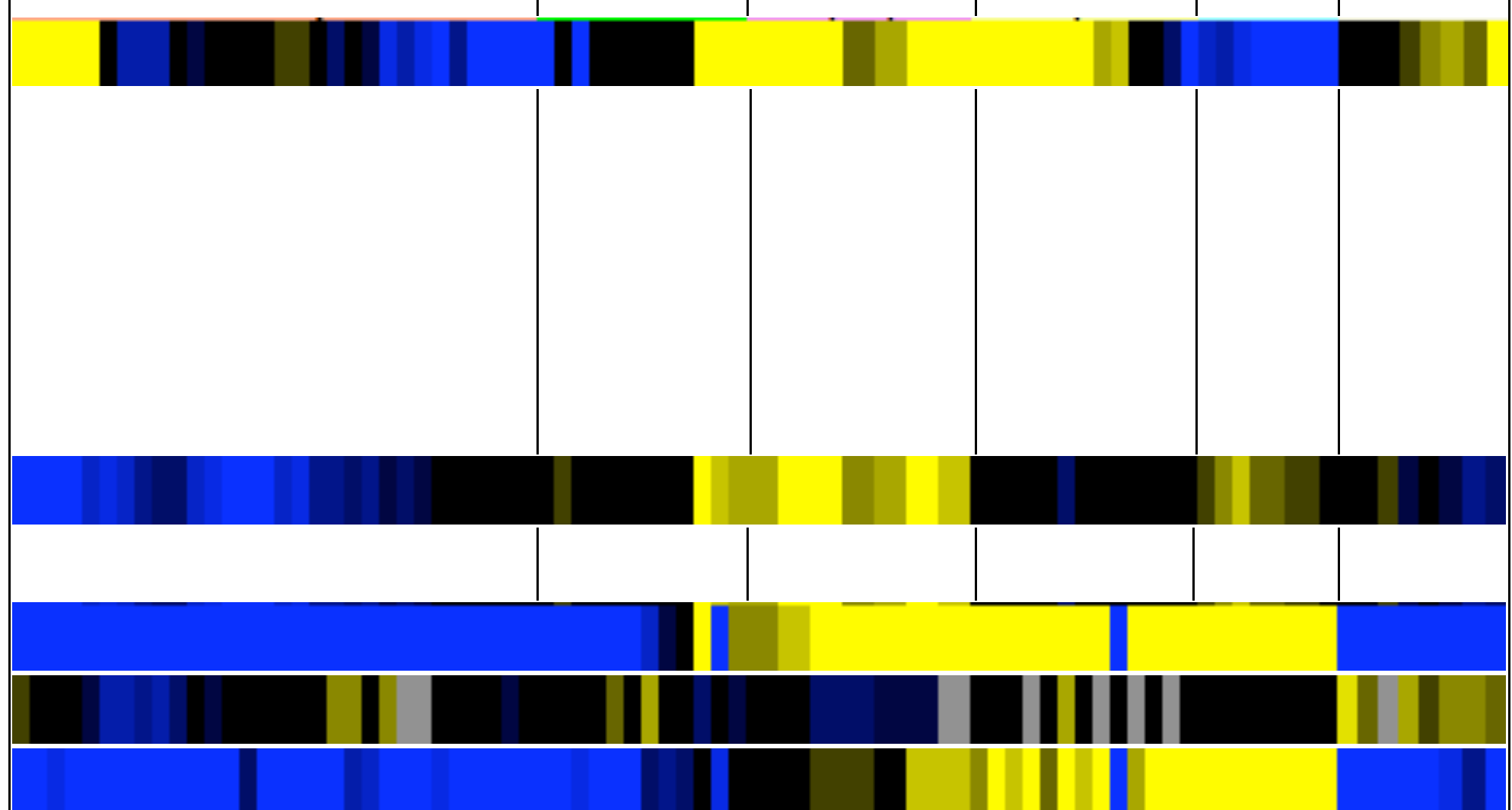

Red: Location in areas known for high recombination (Charlesworth B (1996) Background selection and patterns of genetic diversity in Drosophila melanogaster. Genetical Research 68: 131-149)

Gene Set II. Genes resembling *hsp70* in regulation by polymerase pausing or DNAse hypersensitivity. ID: FlyBase ID; SYM This is the standard abbreviation (gene symbol) for the name of the gene; Location: Chromosome arm and Location of the gene on the cytological (polytene chromosome) map. Genes resembling *hsp70* in regulation by A) polymerase pausing, B) Dnase hypersensitivity, C) high germline transcriptional activity [16], D) dublicated genes. Developmental Gene Expression Timecourse (for color code see above) and Reference.

ID	SYM	Location		Ref.
CG12055	*Gapdh1*	2R-43E17	A	17
CG8893	*Gapdh2*	X-13F17	A	18-21
CG9277	*βTub56D*	2R-56D7-8	A	18
CG10798	*dm*	X-3D2	A	22-25
CG3481	*adh*	(2L)-35B3	A	
CG12181	*Sgs4*	X-3C10	B	26,27
CG6222	*su(s)*	X-1B13	B	28
CG4027	*Act5C*	X-5C7	B	18,29,30
CG4550	*ninaE*	3R-92B4	B	31
CG7939	*RpL32*	3R-99D3	B	32
CG6382	*Elf*	2L-33E4-5	C	
CG8040	*CG32061*	3L-67E3	C	
CG13340		2R-50C5	C	
CG4750		2R-53C7	C	
CG2512	*αTub84D*	3R-84D9	D	33-36
CG1913	*αTub84B*	3R-84R4	D	33-36
CG2238	*Ef2b*	2L-39Ef	D	37
CG11181	*cup*	X-26F5-6	D	38

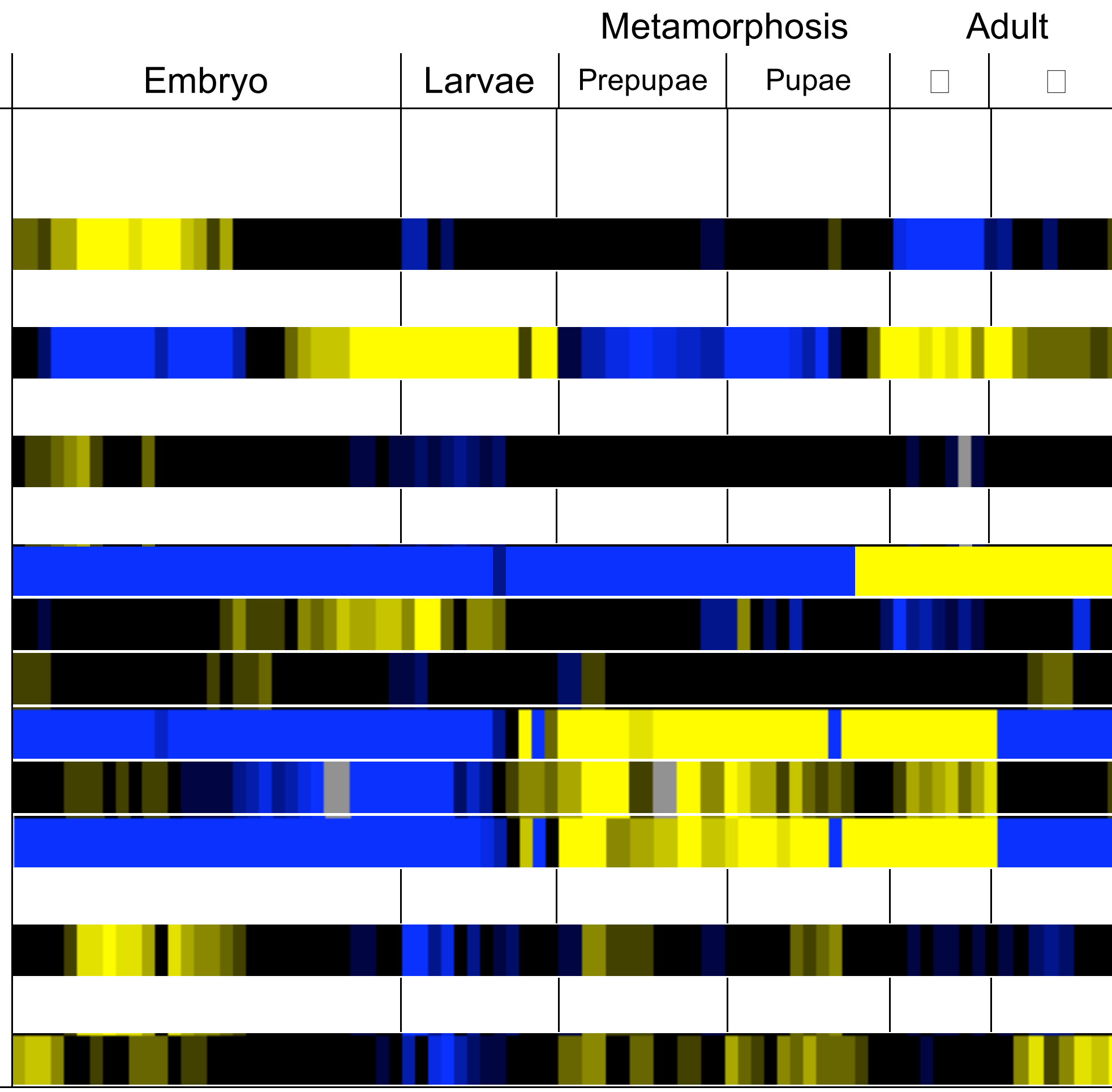

Red: areas known for high recombination (Charlesworth B (1996) Background selection and patterns of genetic diversity in Drosophila melanogaster. Genetical Research 68: 131-149)

Gene Set III. Non-*hsp* genes with limited transcription during the life cycle and negligible germ-line expression (features expected to minimize transposition and its vertical transmission). ID: FlyBase ID; SYM This is the standard abbreviation (gene symbol) for the name of the gene.; Location: Chromosome arm and Location of the gene on the cytological (polytene chromosome) map. Developmental Gene Expression Timecourse (for color code see above) and Reference.

ID	SYM	Location	Ref.
CG7906		3L-70D4	
CG7924		3L-70D4	
CG15634		2L-25A3	
CG12011		3L-62B1	
CG4818		3L-72E2	
CG2916	*Sep5*	2R-43F8	
CG10591		3L-64D5	
CG11650	*Lcp1*	2R-44CR	
CG2961	*Ipod*	X-9B4	
CG9506	*slam*	2L-26C4	
CG7252		3L-68E3	
CG4602	*Srp54*	2L-30E4	
CG9984	*TH1*	X-14C2	
CG14025	*Bsg25D*	2L-25D2-3	
CG10154		3L-70B1	
CG7724		3L-73E5	
CG2070		2R-43E8	
CG6296		3R-97D13	

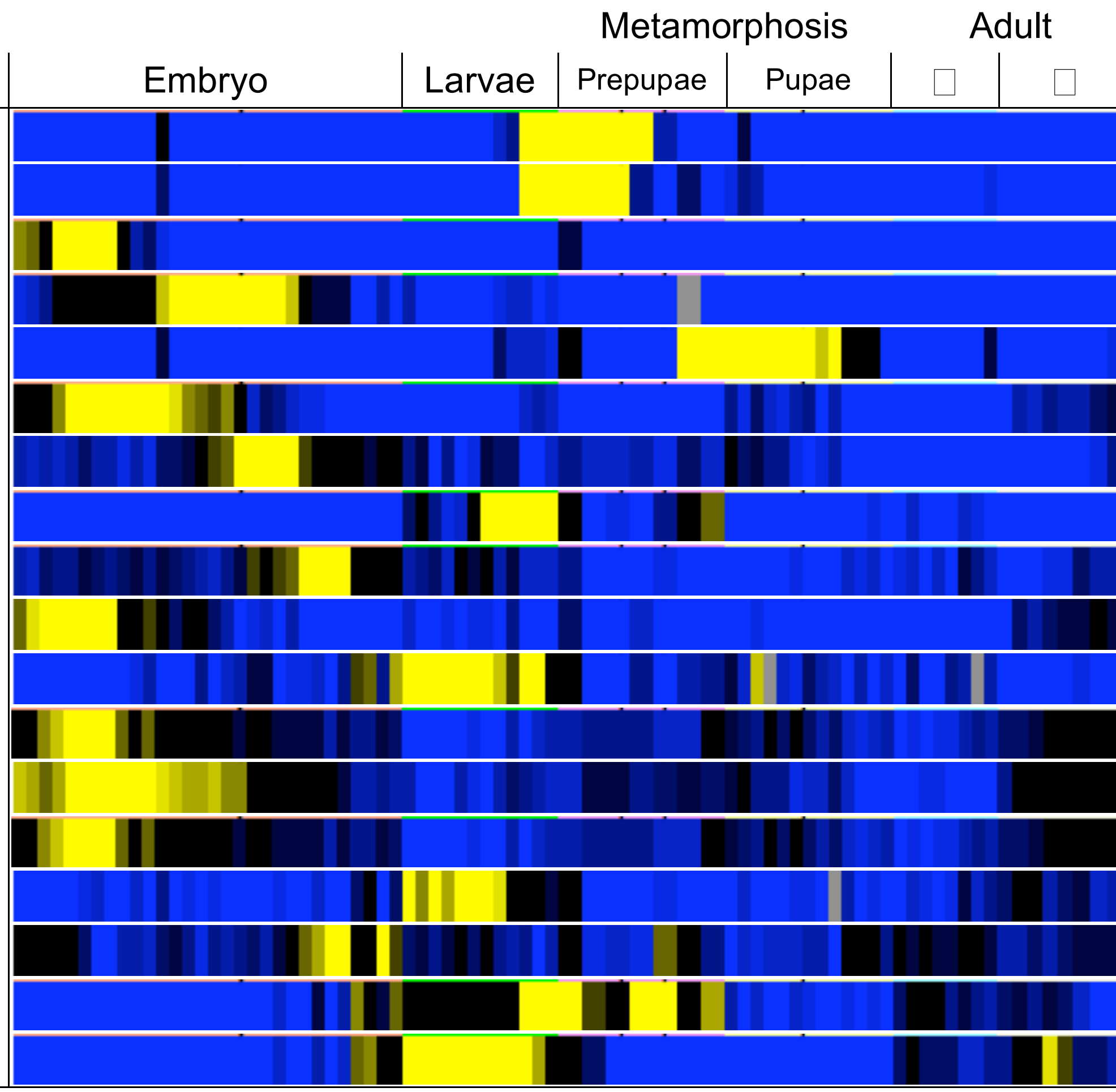

Red: Location in areas known for high recombination (Charlesworth B (1996) Background selection and patterns of genetic diversity in Drosophila melanogaster. Genetical Research 68: 131-149)

Reference List

1. Simon, J.A. & Lis, J.T. A germline transformation analysis reveals flexibility in the organization of heat-shock consensus elements. *Nucleic Acids Research* **15**, 2971-2988 (1987).
2. Klemenz, R. & Gehring, W.J. The control region of the Drosophila heat-shock gene Hsp22. *Experientia* **42**, 649-649 (1986).
3. Klemenz, R. & Gehring, W.J. Sequence requirement for expression of the Drosophila melanogaster heat-shock protein Hsp22-gene during heat-shock and normal development. *Molecular And Cellular Biology* **6**, 2011-2019 (1986).
4. Hultmark, D., Klemenz, R. & Gehring, W.J. Translational and transcriptional control elements in the untranslated leader of the heat-shock gene Hsp22. *Cell* **44**, 429-438 (1986).
5. Bonner, J.J. & Kerby, R.L. RNA polymerase II transcribes all of the heat shock-induced genes of Drosophila melanogaster. *Chromosoma* **85**, 93-108 (1982).
6. Lee, H.S., Kraus, K.W., Wolfner, M.F. & Lis, J.T. DNA sequence requirements for generating paused polymerase at the start of hsp70. *Genes & Development* **6**, 284-295 (1992).
7. Bendena, W.G., Aymesouthgate, A., Garbe, J.C. & Pardue, M.L. Expression of heat-shock locus Hsr-omega in nonstressed cells during development in Drosophila melanogaster. *Developmental Biology* **144**, 65-77 (1991).
8. Marchler, G. & Wu, C. Modulation of Drosophila heat shock transcription factor activity by the molecular chaperone DROJ1. *Embo Journal* **20**, 499-509 (2001).
9. Iliopoulos, I., Torok, I. & Mechler, B.M. The DnaJ60 gene of Drosophila melanogaster encodes a new member of the DnaJ family of proteins. *Biological Chemistry* **378**, 1177-1181 (1997).
10. Kurzikdumke, U., Gundacker, D., Rentrop, M. & Gateff, E. Tumor suppression in Drosophila is causally related to the function of the lethal(2)tumorous imaginal disks gene, a DnaJ homolog. *Developmental Genetics* **16**, 64-76 (1995).
11. Ellis, R.J. & Vandervies, S.M. Molecular chaperones. *Annual Review Of Biochemistry* **60**, 321-347 (1991).
12. Timakov, B. & Zhang, P. The hsp60B gene of Drosophila melanogaster is essential for the spermatid individualization process. *Cell Stress & Chaperones* **6**, 71-77 (2001).

13. Craig, E.A., Ingolia, T.D. & Manseau, L.J. Expression of Drosophila heat-shock cognate genes during heat-shock and development. *Developmental Biology* **99**, 418-426 (1983).
14. Rubin, D.M. et al. Genomic structure and sequence-analysis of Drosophila melanogaster Hsc70 genes. *Gene* **128**, 155-163 (1993).
15. Wilke, N., Sganga, M.W., Gayer, G.G., Hsieh, K.P. & Miles, M.F. Characterization of promoter elements mediating ethanol regulation of hsc70 gene transcription. *Journal Of Pharmacology And Experimental Therapeutics* **292**, 173-180 (2000).
16. Boutanaev, A.M., Kalmykova, A.I., Shevelyou, Y.Y. & Nurminsky, D.I. Large clusters of co-expressed genes in the Drosophila genome. *Nature* **420**, 666-669 (2002).
17. Lis, J. Promoter-associated pausing in promoter architecture and postinitiation transcriptional regulation. *Cold Spring Harbor Symposia on Quantitative Biology* **63**, 347-356 (1998).
18. O'Brien, T., Hardin, S., Greenleaf, A. & Lis, J.T. Phosphorylation of RNA polymerase II C-terminal domain and transcriptional elongation. *Nature* **370**, 75-77 (1994).
19. Rasmussen, E.B. & Lis, J.T. Short transcripts of the ternary Complex provide insight into RNA Polymerase II elongational pausing. *Journal of Molecular Biology* **252**, 522-535 (1995).
20. Rougvie, A.E. & Lis, J.T. Postinitiation Transcriptional Control In Drosophila-Melanogaster. *Molecular And Cellular Biology* **10**, 6041-6045 (1990).
21. Sun, X.H., Lis, J.T. & Wu, R. The Positive And Negative Transcriptional Regulation Of The Drosophila Gapdh-2 Gene. *Genes & Development* **2**, 743-753 (1988).
22. Krumm, A., Meulia, T., Brunvand, M. & Groudine, M. The block to transcriptional elongation within the human c-myc gene is determined in the promoter-proximal region. *Genes & Development* **6**, 2201-2213 (1992).
23. Plet, A., Eick, D. & Blanchard, J.M. Elongation and premature termination of transcripts initiated from c-fos and c-myc promoters show dissimilar patterns. *Oncogene* **10**, 319-328 (1995).
24. Spencer, C.A. & Groudine, M. Transcription elongation and eukaryotic gene-regulation. *Oncogene* **5**, 777-785 (1990).
25. Strobl, L.J. & Eick, D. Hold back of RNA Polymerase II at the transcription start site mediates down-regulation of c-myc in vivo. *Embo Journal* **11**, 3307-3314 (1992).

26. McGinnis, W., Shermoen, A.W. & Beckendorf, S.K. A transposable element inserted just 5' to a Drosophila glue protein gene alters gene-expression and chromatin structure. *Cell* **34**, 75-84 (1983).
27. Shermoen, A.W. & Beckendorf, S.K. A complex of interacting DNase I-hypersensitive sites near the Drosophila glue protein gene, Sgs4. *Cell* **29**, 601-607 (1982).
28. Voelker, R.A., Graves, J., Gibson, W. & Eisenberg, M. Mobile element insertions causing mutations in the Drosophila suppressor of sable locus occur in DNase-I hypersensitive subregions of 5'-transcribed nontranslated sequences. *Genetics* **126**, 1071-1082 (1990).
29. Chung, Y.T. & Keller, E.B. Positive and negative regulatory elements mediating transcription from the Drosophila melanogaster actin-5c distal promoter. *Molecular And Cellular Biology* **10**, 6172-6180 (1990).
30. Chung, Y.T. & Keller, E.B. Regulatory elements mediating transcription from the Drosophila melanogaster actin 5C proximal promoter. *Molecular And Cellular Biology* **10**, 206-216 (1990).
31. Picking, W.L. et al. Control of Drosophila opsin gene expression by carotenoids and retinoic acid: Northern and western analyses. *Experimental Eye Research* **63**, 493-500 (1996).
32. Wong, Y.C., Oconnell, P., Rosbash, M. & Elgin, S.C.R. DNase I hypersensitive sites of the chromatin for Drosophila melanogaster ribosomal protein 49 gene. *Nucleic Acids Research* **9**, 6749-6762 (1981).
33. Theurkauf, W.E., Baum, H., Bo, J.Y. & Wensink, P.C. Tissue-specific and constitutive alpha-Tubulin genes of Drosophila melanogaster code for structurally distinct proteins. *Proceedings Of The National Academy Of Sciences Of The United States Of America* **83**, 8477-8481 (1986).
34. Mischke, D. & Pardue, M.L. Organization and expression of alpha-Tubulin genes in Drosophila melanogaster - One member of the alpha-Tubulin multigene family is transcribed in both oogenesis and later embryonic-development. *Journal Of Molecular Biology* **156**, 449-466 (1982).
35. Kalfayan, L. & Wensink, P.C. Alpha-Tubulin genes of Drosophila. *Cell* **24**, 97-106 (1981).
36. Mischke, D. & Pardue, M.L. Organization and expression of alpha-Tubulin genes in Drosophila melanogaster. *European Journal Of Cell Biology* **22**, 14-14 (1980).

37. Grinblat, Y., Brown, N.H. & Kafatos, F.C. Isolation and characterization of the Drosophila translational elongation factor-II Gene. *Nucleic Acids Research* **17**, 7303-7314 (1989).

38. Wilhelm, J.E., Hilton, M., Amos, Q. & Henzel, W.J. Cup is an elF4E binding protein required for tooth the translational repression of oskar and the recruitment of Barentsz. *Journal Of Cell Biology* **163**, 1197-1204 (2003).
