# Supplementary material for: Heat-Shock Promoters: Targets for Evolution by P Transposable Elements in Drosophila
Source: PLoS Genet. 2006 Oct 6;2(10):e165. doi: 10.1371/journal.pgen.0020165 (PMC1592238; doi:10.1371/journal.pgen.0020165)
Supplement: Supplementary file 2 [file pgen.0020165.st002.pdf]

Table S2: UFW Primers for Gene Set I

SET	Primer	Sequence 5'-3'	Length [bp]	Tm [°C]
1	Hsp22_1a	CCAgCACCTTgACCTTTAgC	20	59.88
1	Hsp22_2a	CgggCggCTCgTggAAgAAg(N)10	30	
1	Hsp22_3a	gACgTCCAgggTgAgTTTgT	20	60.01
1	Hsp22_4a	CgTgAAAgggCgAggAgAgg	20	66.63
1	Hsp22_5a	ATCggTAAggAACgCATTgT	20	59.46
1	Hsp22_1b	ggAggAAgTgCCTggAgCTA	21	61.82
1	Hsp22_2b	gATTTTCCCTCCACCAggAC(N)10	30	
1	Hsp22_3b	CCTTgACCTTTAgCTCgCTgT	21	60.93
1	Hsp22_4b	gAgCgAACTCCTgCTCCTg	19	61.25
1	Hsp22_5b	CAATCTgCTgCCAgTTCCTC	20	60.94
1	Hsp22_6b	CCTTgACCTTTAgCTCgCTgT	21	60.93
1	Hsp22_7b	gTggAAgAAggCgTgAAAgg	20	62.07
1	Hsp23_1a	CAgAgCATAgCggCggACAA	20	66.86
1	Hsp23_2a	TCCTTTCCgATTTTCgACAC(N)10	30	
1	Hsp23_3a	ACggAgTTgTCCTgCACTTT	20	59.77
1	Hsp23_4a	TgCTgCTCCATCggTCCAAC	20	66.91
1	Hsp23_5a	ggCAAggCTCAACAACAATg	20	61.61
1	Hsp23_1b	CCTgCACTTTgACCACCAgT	20	61.16
1	Hsp23_1b	gTCCAACCAgggCCAAgTAg(N)10	25	
1	Hsp23_1b	gACCTggAAgCCATCCTTTC	20	60.97
1	Hsp23_1b	CTCTggCgCTggCAgTAgTA	20	61.65
1	Hsp23_1b	ggCTCATAgAAgggCACCAT	20	61.38
1	Hsp23_1b	gCCCACCTgTTTCTCCAg	18	59.18
1	Hsp23_1b	ggCAAggCTCAACAACAATg	20	61.61
1	Hsp26-1a	ACCAAgATggAgTCgTCCAC	20	59.97
1	Hsp26-2a	TgTTCCTTTTgCgAgATTgA(N)10	30	
1	Hsp26-3a	ATCCACAAgCgAAAgCAgAg	20	60.54
1	Hsp26-4a	TCTgTgCTCTgTTCgTCAAg	20	57.67
1	Hsp26-5a	TACCCgCTggAgCTTTTCTA	20	59.97
1	Hsp26-1b	CATTCCCgCTACgTgCTg	18	61.40
1	Hsp26-2b	TgTTCCTTTTgCgAgATTgA(N)10	25	
1	Hsp26-3b	CCCAgTCCAAgCTCgTAgAT	20	59.31
1	Hsp26-4b	TCTgTgCTCTgTTCgTCAAg	20	57.67
1	Hsp26-5b	TACCCgCTggAgCTTTTCTA	20	59.97
1	Hsp26-6b	ATCCACAAgCgAAAgCAgAg	20	60.54
1	Hsp27-1	AAgTgACgCTggATCATTCC	20	60.08
1	Hsp27-2	ACACCTggAAgCCATCTTTg(N)10	30	
1	Hsp27-3	CTCCTCTCgTACggCgAATA	20	60.37
1	Hsp27-4	CAgggTgTTgggCAgTAgCA	20	61.67
1	Hsp27-5	CCAgTCggTgCggTAgTCAT	20	59.72
1	Hsp27-6	gggCAgCAgAgCgTTTggAC	20	60.72
1	Hsp27-7	TTgAACTgCgACACATCCAT	20	60.12

1	Hsp68_1	CTgTTggCTCgTTgATgATg	20	60.26
1	Hsp68_2	gAACggCCAgTgCTTAATgT(N)10	25	
1	Hsp68_3	CTTgAACTCCACgCTTATCT	20	54.70
1	Hsp68_4	ATCTTggAATCgTCgAAACg	20	60.07
1	Hsp68_5	ggCTggCATCTTggCTATTT	20	61.46
1	Hsp68_6	ATCTTTggTTTgCCgTTgTC	20	59.98
1	Hsp70Ab_1	CTCCTCgggggCAAATCTCT	20	63.02
1	Hsp70Ab_2	TCTTggggTCgTCgTATTTT(N)10	30	
1	Hsp70Ab_3a	CgTCgCTTACCACCTTgAAA	20	61.18
1	Hsp70Ab_3b	CACTgTgTTTCTggggTTCA	20	59.57
1	Hsp70Ab_4	ACTgTgTTTCTggggTTCAT	20	56.9*
1	Hsp70Ab_5	AgACACCCACgCAggAgTAg	20	60.32
1	Hsp70Ab_6	TCATgTCCTCTgCgATCTTg	20	59.94
1	Hsp83-1a	CTTCCTgATTgACgCTTTAC	20	55.12*
1	Hsp83-1b	TCAATTTgATgCCCAATgAA	20	59.87
1	Hsp83-2	CCATCCACgAATCCCAgAgg(N)10	30	
1	Hsp83-3a	CgCTTgTTgATgTTgTAgAA	20	54.95*
1	Hsp83-3b	gTTTTgAggCATAgCCgAAg	20	59.89
1	Hsp83-4a	TAggTgTTgCTgCTTTggAg	20	59.07
1	Hsp83-5a	ACCCAATCTTTCAACCTCTT	20	55.29*
1	Hsp83-5b	gAAgAgCTTCACCgTTCTgg	20	59.99
1	Hsp83-6	CTTCgTCggCTTCgTCTTTC	20	62.33
1	Hsrw-1a	gCACACATCCTTACATCCTg	20	56.51*
1	Hsrw-1b	AgAAAACTgCCTgCTgCATT	20	60.02
1	Hsrw-2	TTgTTTCCAACTCgAgAgCA (N)10	30	
1	Hsrw-3	CTgACCAgCAAAATCgATgA	20	59.80
1	Hsrw-4	AAATACTACgTgggCCCTTg	20	58.97
1	Hsrw-5	ACTCgTACTgCTgCTgCTCg	20	61.89
1	Hsrw-6	ACATCTTTTCCAgggggTTT	20	59.67
1	Hsrw-7a	gggACACgATTTTTACACTT	20	54.22*
1	Hsrw-7b	CTgCTgggACACgATTTTTA	20	58.77
1	DnaJ-1-1	AgCACCTTCTCCTCCTTgTA	20	56.57
1	DnaJ-1-2	CCCTTCAATCCATCCTCACC (N)10	30	
1	DnaJ-1-3	ATgCATCCCTTgTCCACTTC	20	59.93
1	DnaJ-1-4	CCTgTgggCTCTTgTTCTTg	20	60.82
1	DnaJ-1-5a	gAgTgCCAgTTTgCggTAgg	20	63.05*
1	DnaJ-1-5b	AgTTTgCggTAggCCTTCTT	20	60.26
1	DnaJ-1-6	CCAAAgAACTgggCAAATgT	20	59.97
1	DnaJ-1-7	ATCgCCgTggAACTggTAAg	20	62.33
1	DnaJ_60-1	CggCTTTATCAgggTCTTgT	20	59.19
1	DnaJ_60-2	gAAAgggCAgggTTTAggAg (N)10	30	
1	DnaJ_60-3	gCCTCggAgATCTgAACAAA	20	60.34
1	DnaJ_60-4	gAgAgCTggACAAAggCATT	20	59.43
1	DnaJ_60-5	ACTTCACgggTgCTgCAAT	19	61.69
1	DnaJ_60-6	gACACgCAgCATTgCTCTTA	20	60.17
1	DnaJ_60-7	gTTTTCTTggTTTgTCgTTC	20	54.83
1	DnaJ-1	CgTggCgTAgTAgTCCTTgg	20	60.70
1	DnaJ-2	ACAACATgCCgTAACATCCA(N)10	30	

1	DnaJ-3	TggAgAggATTCggTAggAg	20	59.23
1	DnaJ-4	gTgCgTAgCgAgCgAAAATA	20	61.41
1	DnaJ-5	ggCgTgAAAgCgAATgAAAT	20	62.37
1	DnaJ-6a	CTgCCggAACACgAATAAT	19	58.59
2	DnaJ-6b	gCTgCCggAACACgAATAAT	20	62.26
1	CG9920-1a	TATggCAgTgTgAgTggTAg	20	54.6*
1	CG9920-1b	CggATgAAgTgTgTggAgTg	20	60.15
1	CG9920-2	CTCCTTgggCACCgACTCCT(N)10	30	
1	CG9920-3	gCAATTCggAAgCAAAATgA	20	61.09
1	CG9920-4	ggTggTCTTCACCTCgAAAC	20	59.55
1	CG9920-5	CCTCgAAACgCTggATTAgA	20	60.34
1	CG9920-6	AACATACTCgCgCTTgTCgT	20	60.86
1	CG9920-7a	CCTTAgTTCCACCgTATTTg	20	54.87*
1	CG9920-7b	ggACAAgTgTCCAgCTCCAg	20	60.86
1	Hsp60-1	ATCTCCTCgggCgTgCTAAC	20	63.82
1	Hsp60-2	CgCCgTCCTTggTgATTTTg(N)10	30	
1	Hsp60-3	CgCgACATggTCTTAAggTT	20	60.13
1	Hsp60-4a	ACgCACATCCTTggCATAgC	20	63.42*
1	Hsp60-4b	AACgCACATCCTTggCATAg	20	61.05
1	Hsp60-5	CTAATggAggAgCgAgCAAg	20	60.11
1	Hsp60-6	gATCTTCTCgAAgCCCTCCT	20	59.92
1	Hsp60-7	CgAAACTggCAAACggAACA	20	64.33
1	Hsp60b_1a	CggAgAgggTggCTACTTgT	20	61.60
1	Hsp60b_2a	ggATTAgCCCCCATggTAAT(N)10	30	
1	Hsp60b_3a	ACACCTCgACgAATCTCCAC	20	60.12
1	Hsp60b_4a	CgTATTATCggCCACATCCT	20	59.81
1	Hsp60b_5a	ggTCACAgCAACggCATCAg	20	65.17
1	Hsp60b_1b	TgAATCCTTCCTTggCAATC	20	60.01
1	Hsp60b_2b	gTCgCTCCACgATCACACT(N)10	29	
1	Hsp60b_3b	CgTATTATCggCCACATCCT	20	59.81
1	Hsp60b_4b	ACggCATCAgCCAgAATATC	20	60.07
1	Hsp60b_5b	ggATCATCATTgCCCgTACT	20	59.78
1	Hsp60b_6b	CATgTgCTggTCCTTTAggg	20	60.51
1	Hsp60b_7b	gCgAAACAgCgACTAgAggT	20	59.64
1	Hsc70_2-1	CATCTTggCATCgCTCAgTg	20	62.94
1	Hsc70_2-2	gCTCTggCATTTCCCTTgAT(N)10	30	
1	Hsc70_2-3	ggAAgAggTCCATgTTCAgC	20	59.66
1	Hsc70_2-4	AAATCCTCACCACCCAAATg	20	59.65
1	Hsc70_2-5a	AggTgCCgCCgCCCAAgTC	19	72.16*
1	Hsc70_2-5b	CTTCACCTCgAACAgggAAC	20	59.70
1	Hsc70_2-6	ggCATCAATCTCCAAggATg	20	60.43
1	Hsc70_2-7	CCTTgCCACCCAgATAgACC	20	61.80
1	Hsc70_3-1a	TggCTgTTCggTgggTgACT	20	66.31
1	Hsc70_3-1b	ATgAggAgACTggCTgTTCg	20	60.41
1	Hsc70_3-2	CgAACATCTggACgAAATCT(N)10	30	
1	Hsc70_3-3	TgCggCTATCgTTTgCTATg	20	62.17
1	Hsc70_3-4	CgTACgTggTTAATCgTCTgC	21	60.57
1	Hsc70_3-5	ggCgATCCATCTgAAATTgT	20	59.90

1	Hsc70_3-6a	CTCgTCCACCACCCAACTgA	20	64.52*
1	Hsc70_3-6b	CAgCTgAgCgATgACATTCT	20	59.14
1	Hsc70_3-7a	CTgTCCATTTgCggggTgAg	20	66.08
1	Hsc70_3-7b	ACTCAAgTgCCAggATACgg	20	60.13
1	Hsc70_4-1a	TTgACTACCACTCgCATCTT	20	55.92
1	Hsc70_4-1b	TCTTGATGCCATCCTCGAAT	20	60.57
1	Hsc70_4-2	TTCTCgTTgACATCCTCCAg (N)10	30	
1	Hsc70_4-3	CCCATTTCCggAggTCTg	18	61.43
1	Hsc70_4-4a	ATCCgAAgTTTTgCTAAgAA	20	54.39*
1	Hsc70_4-4b	TCGACGCTTCAAAGATTGTG	20	59.99
1	Hsc70_4-5	gCTgTTggATgCCCTTgTTT	20	62.33
1	Hsc70_4-6	CgCCAgCgTTTTgAATACTT	20	60.26
1	Hsc70_4-7	CTgAAggTCCCCgAgTATCC	20	60.84
1	Hsc70_5-1	ATCCAgCCCTTgTTgAgCAg	20	62.66
1	Hsc70_5-2	CTCCTCCCgAATCTCATCC (N)10	30	
1	Hsc70_5-3	ACCATTgCCATTggATgAgT	20	60.20
1	Hsc70_5-4	gTAgTCCCTTCTCgCCAACC	20	61.02
1	Hsc70_5-5a	ATCTCAgCCATTTTCTTgC	19	55.43*
1	Hsc70_5-5b	TGTTGGGGATCAGAGAGGAC	20	60.05
1	Hsc70_5-6	AAgATCTCCgTggCATTCAC	20	60.08
1	Hsc70_5-7	gATTggTCCgCTgCTgTAgA	20	61.35
1	Hsp67Ba-1a	TCTTgACggTCAgTTCgTTg	20	59.87
1	Hsp67Ba-2a	CACgATTgTACggATgATgg(N)10	30	
1	Hsp67Ba-3a	CCACggAgTAggCggATTTg	20	64.93
1	Hsp67Ba-4a	CCgCCgACTgACCCTTgATg	20	68.39
1	Hsp67Ba-5a	ATTgAAATCgTgCAgCTCCT	20	59.84
1	Hsp67Ba-1b	CAATTCCTTTTCCAGCTCCA	20	60.18
1	Hsp67Ba-2b	AGCTGTGGCAACTGTGAGGT(N)10	30	60.93
1	Hsp67Ba-3b	CACGATTGTACGGATGATGG	20	59.80
1	Hsp67Ba-4b	CCAGTGGATACAACCCGAAT	20	59.67
1	Hsp67Ba-5b	ATTGAAATCGTGCAGCTCCT	20	59.84
1	Hsp67Ba-6b	CCGACTGACCCTTGATGG	18	60.04
