# Supplementary material for: Heat-Shock Promoters: Targets for Evolution by P Transposable Elements in Drosophila
Source: PLoS Genet. 2006 Oct 6;2(10):e165. doi: 10.1371/journal.pgen.0020165 (PMC1592238; doi:10.1371/journal.pgen.0020165)
Supplement: Supplementary file 3 [file pgen.0020165.st003.pdf]

**Table S3: UFW Primers for Gene Set II**				
SET	Primer	Sequence 5'-3'	Length [bp]	Tm [°C]
2	gapdh1_1a	TCCgTTAATTCCgATCTTCg	20	60.03
2	gapdh1_2a	ACTgCCgCTTTTTCCTTTTC(N)10	30	
2	gapdh1_3a	TggCTgAgTTCCTgCTgTCT	20	61.15
2	gapdh1_4a	AAATTAggAgAATggCgCTg	20	59.32
2	gapdh1_5a	ggggAgggTTgATgAgTATT	20	57.77
2	gapdh1_1b	gTgAACACTCCggTggACTC	20	60.57
2	gapdh1_2b	CCgTgAgTCgAgTCgAATTT(N)10	30	
2	gapdh1_3b	ACATACTCggCTCCAgCACT	20	59.90
2	gapdh1_4b	gACATCgATgAAgggATCgT	20	59.89
2	gapdh1_5b	TCCgTTAATTCCgATCTTCg	20	60.03
2	gapdh1_6b	TgAACACggTgATCTTCTgg	20	59.68
2	gapdh2-1	gCCAgAggAgCCAAgCAgTT	20	64.19
2	gapdh2-2	gTgATCTTCTggCCgTTgAC(N)10	30	
2	gapdh2-3	TCAAgTgAgTggATgCCTTg	20	59.83
2	gapdh2-4	gggTCgAATCgAACTTgAAC	20	59.53
2	gapdh2-5	AgTTCACATCgATgAAgggA	20	58.07
2	gapdh2-6	TggACTCCACgATgTATTCg	20	59.52
2	gapdh2-7	gATgCgACCAAATCCATTg	19	59.87
2	βTub56D-1	AggTCACCgTATgTgggTgT	20	60.16
2	βTub56D-2	CATgATCCTgTCggggTACT (N)10	30	
2	βTub56D-3	TgTCgATgCAgTAggTCTCg	20	60.01
2	βTub56D-4	CggATCTTggAAATCAgCAg	20	60.73
2	βTub56D-5	TgTgTgAgTTggAAgCCTTg	20	59.87
2	βTub56D-6	gACggCACAACCgAgTATg	19	60.13
2	D-Myc-1a	gTCCTggTTgTTCCgCTTgC	20	65.26*
2	D-Myc-1b	gggCggTATTAAATggACCT	20	60.04
2	D-Myc-2	TgTTgATTTgACTgggTgTT (N)10	30	
2	D-Myc-3	CgCTTCTgACAgACCgTgTA	20	60.05
2	D-Myc-4a	CgCTgTAgAgATTCgTAgAg	20	52.95*
2	D-Myc-4b	TCgTCTgCgATCgTTgATAC	20	59.83
2	D-Myc-5a	CCgCTCTCTATTCCATTACC	20	56.41*
2	D-Myc-5b	TgCACTgTTTCCgCTCTCTA	20	59.74
2	D-Myc-6	CAgATCTgCgAgCTCCTTCT	20	59.85
2	D-Myc-7	AATggTggACgACgAAAggA	20	62.76
2	Adh-1	gggCACggTCACATCATAgg	20	63.63
2	Adh-2	ACCAATgCCTCCCAgACC (N)10	28	
2	Adh-3	CggTCACCTTTggATTgATT	20	59.79
2	Adh-4	TCACgTTCTTgTTggTCAAAg	21	58.81
2	Adh-5	TTAgCAggCTCTTTCgATCTg	21	59.74
2	Adh-6	ggTCgAggATCACCAggTT	19	59.91
2	Adh-7	ggAAgAgggCTCCgTTAgTT	20	59.71
2	Sgs4_1a	CATCTCggTggTTCggTTTC	20	62.67

2	Sgs4_1b	CATCTTggTggTTCggTTTC	20	60.35
2	Sgs4_2	CCAATAACACAACCAACAAC(N)10	30	
2	Sgs4_3a	TCggTggTTCAgTTTTACAg	20	56.27*
2	Sgs4_3b	TCggTggTTCAgTTTTACAgC	21	60.16
2	Sgs4_4	ACTCCAAgCgCATCTTgACT	20	60.02
2	Sgs4_5	CATTTTgCCATCgACACATC	20	59.93
2	Sgs4_6	gTAgAACCTgACggggCAAg	20	61.96
2	Su(s)-1	AgCgATATgCggCAAgTAgT	20	59.90
2	Su(s)-2	TAggTggCATCCgAgTggT (N)10	30	
2	Su(s)-3	TCCACgAgATgTCTTCTCCA	20	59.34
2	Su(s)-4a	ATAAAgCAgAAAgCAgAgg	19	52.41*
2	Su(s)-4b	gTCgCCATAAAgCAgAAAgC	20	59.99
2	Su(s)-5	TTTgggCAAAgTTCATgTCA	20	60.09
2	Su(s)-6	CACTTTgCgggAAgTCTgAT	20	60.25
2	Su(s)-7	CgATAAATTgCACTCgCTgA	20	59.98
2	Act5C-1	ggTCATCTTCTCACggTTgg	20	60.51
2	Act5C-2	CAgTTggTCACgATACCgTg (N)10	30	
2	Act5C-3	CACACgCAgCTCATTgTAgA	20	58.60
2	Act5C-4	AggATACCACgCTTgCTCTg	20	60.42
2	Act5C-5	CACAATCgATgggAAgACg	19	60.06
2	Act5C-6	AggTgTggTgCCAgATCTTC	20	60.12
2	Act5C-7	gAgCAgCAACTTCTTCgTCA	20	59.32
2	NinaE_1a	TTggTggTggCgAATATgTA	20	59.81
2	NinaE_2a	AggAAATCATgCCgATCATg(N)10	30	
2	NinaE_3a	CCACgCCATTTCCgCACCAg	20	71.16*
2	NinaE_4a	CATgTAggCggTCAggATCT	20	60.10
2	NinaE_5a	TACTgCAAAgCTgCAACACC	20	60.06
2	NinaE_1b	ATCATgggCgTgTTTgTgAT	20	61.20
2	NinaE_2b	CATgTAggCggTCAggATCT(N)10	30	
2	NinaE_3b	gAgATggCCAggTTgATgAC	20	60.48
2	NinaE_4b	ggTTCCAgTACgggCTgAT	19	59.94
2	NinaE_5b	CTTgTCCACCACCgATCC	18	59.88
2	NinaE_6b	TTggTggTggCgAATATgTA	20	59.81
2	RpL32_1	gACAATCTCCTTgCgCTTCT	20	59.58
2	RpL32_2	gTTCgATCCgTAACCgATgT(N)10	30	
2	RpL32_3	ACgTTgTgCACCAggAACTT	20	60.61
2	RpL32_4a	CTggTggCggATgAAgTgCT	20	65.89
2	RpL32_4b	AgCATATCgATCCgACTggT	20	59.54
2	RpL32_5a	CTgTATgCTgggCggATggT	20	65.48
2	RpL32_5b	TCACgATCTTgggCCTgTAT	20	60.48
2	RpL32_6	CTCTgTTgTCgATACCCTTgg	21	60.61
2	Elf-1a	ATTTTgTCCgAggggTTgAg	20	61.24
2	Elf-2a	TgCAgTTggAggCggTggAg (N)10	30	
2	Elf-3a	CATCgATgggCgTTggAT	18	62.88
2	Elf-4a	CgACggAATCgggTgTgg	18	65.93
2	Elf-5a	TggTggCTggCgTggCTgAA	20	72.75
2	Elf-6a	ggAATTggAggATgATgTgg	20	60.13
2	Elf-7a	ACTACTgCCgCCgCCTCCTC	20	68.24

2	Elf-1b	gCTgTgCTgAgggTgCTg	18	61.85
2	Elf-2b	CCgCCTCCTCAgCTACATT	19	
2	Elf-3b	gACggAATCgggTgTggT	18	61.81
2	Elf-4b	ACTgggCACAAATTCTACCg	20	59.99
2	Elf-5b	TCACgTTCAgCgTTgAgAAC	20	60.03
2	Elf-6b	AgCCgCTgCAACCACTACT	19	61.01
2	CG31061-1a	CCACCgATgAAAgCAgAgCA	20	64.73
2	CG31061-1b	CCCggTgTACCgATAATgAC	20	60.07
2	CG31061-2	AgATTAgAgTTTTgCCAgTT (N)10	30	
2	CG31061-3	CCgAAACgATTTgCAgAgAT	20	60.21
2	CG31061-4	CAgCTgCCTgAATCTTCgTT	20	60.54
2	CG31061-5	AgACgCAgTTCCTCgAAggT	20	61.36
2	CG31061-6	CATgCggTTggTTTATgTTg	20	59.85
2	CG31061-7	CACATCgCTACTTCgTTCCT	20	57.98
2	CG13340-1	TTgAAgCCgATgCCCTCCAg	20	60.29
2	CG13340-2	gACgCgCTggTCAATCTT (N)10	28	
2	CG13340-3	CgATgTTgTggAACACCTTg	20	60.00
2	CG13340-4a	CgCCCTTCgCCATCgTCTTg	20	70.38*
2	CG13340-4b	gAgTCAgCTTgggATCCTTg	20	59.80
2	CG13340-5a	gCCATCgCCgCAggAgAATC	20	69.56*
2	CG13340-5b	gAgAATCTCACgCCAgCACT	20	59.52
2	CG13340-6a	TCACAgATCgCCTTCATCAg	20	59.94
2	CG13340-6b	ACAgATCgCCTTCATCAgCT	20	59.98
2	CG13340-7	CACACgCATTTTTCAACgAg	20	60.29
2	CG4750-1	TCgTCCAgTTCCTCgTTTTC	20	60.23
2	CG4750-2	CAgAgCATCATCCAAggTCA (N)10	30	
2	CG4750-3	CAgACCCTTTCCgggAgT	18	60.04
2	CG4750-4	gCTTATCgCCATCCTTggTA	20	60.06
2	CG4750-5	CACAACACCCTTgggACTCT	20	60.00
2	CG4750-6	gTTCACggATCAgggTCAAC	20	60.37
2	CG4750-7	gATTgTCCAgAACgATTTTg	20	55.64
2	αTub84D-1a	AACgAgTggAAgATgAggAA	20	57.29
2	αTub84D-1b	ggCCCACgTggATAgAgATA	20	59.92
2	αTub84D-2	AgCTCCAgTCTCgCTgAAgA (N)10	30	
2	αTub84D-3	ggTggAACAgCTgACggTA	19	59.69
2	αTub84D-4	ggTgTTAAACgAgTCATCACCT	22	58.11
2	αTub84D-5a	TCTTgTCggAgggCATCTgg	20	65.87*
2	αTub84D-5b	gTCTTgTCggAgggCATCT	19	60.22
2	αTub84D-6	gTTCCAgTACggACCTCgTC	20	59.58
2	αTub84D-7	CTCCCAgCAggCATTTCCAA	20	65.67
2	αTub84B_1a	CTCgCTgAAgAAggTgTTgA	20	59.16
2	αTub84B_2a	CCAgCAggCgTTTCCAATCT(N)10	30	
2	αTub84B_3a	CCAAgCAgTAgAgCTCCCAg	20	60.15
2	αTub84B_4a	gATgAggAggAAgggAAAAC	20	57.62
2	αTub84B_5a	TTACACACCCCAAgAAAATCg	21	59.85

2	αTub84B_1b	CCgCATCCTCCTTACCAgT	19	60.08
2	αTub84B_2b	CCAgTCTCgCTgAAgAAggT(N)10	30	
2	αTub84B_3b	gTggAACAgCTgACggTAgg	20	60.71
2	αTub84B_4b	AgCAgTAgAgCTCCCAgCAg	20	59.91
2	αTub84B_5b	AATCTggACACCAgCCTgAC	20	60.12
2	αTub84B_6b	gACCACAgTgggTTCCAgAT	20	59.82
2	Ef2b_1a	ATCCTTTTCCTCCACCTCAA	20	58.57
2	Ef2b_2a	AAgggAATCggTCAgAgTgg(N)10	30	
2	Ef2b_3a	TCgTCCTTgCgggTgTCAgT	20	66.43*
2	Ef2b_4a	TCTACgTgggCAATCACAgA	20	60.26
2	Ef2b_5a	gATgTTCCgCTTCTTgTCCA	20	61.19
2	Ef2b_1b	CACCAgATCCTTTTCCTCCA	20	60.04
2	Ef2b_2b	TgATACCAgCCTTCgACACA(N)10	30	
2	Ef2b_3b	ggTAATgCAgCgCTCCTg	18	60.53
2	Ef2b_4b	CAgACATgTTgCggATgTTC	20	60.12
2	Ef2b_5b	gATCTCgTCgACggTgAAgT	20	60.27
2	Ef2b_6b	AACgAgTCTCACCAgCCTTg	20	60.44
2	cup-1	CAgCTTgAggTAgCCCAgTC	20	60.01
2	cup-2	TggAgTTggTggCggTggAg (N)10	30	
2	cup-3	TCCTCCCACTTCTCggAgTT	20	61.16
2	cup-4	ACTCgAACTTgCCCTTCTCA	20	59.99
2	cup-5	AgCACCTCATCCTgCTgATC	20	60.38
2	cup-6	gTCgTCATgCTCTTgACTgg	20	59.42
2	cup-7	AATTCgATCgCCTCCTCTTT	20	60.17
