# Supplementary material for: Heat-Shock Promoters: Targets for Evolution by P Transposable Elements in Drosophila
Source: PLoS Genet. 2006 Oct 6;2(10):e165. doi: 10.1371/journal.pgen.0020165 (PMC1592238; doi:10.1371/journal.pgen.0020165)
Supplement: Supplementary file 4 [file pgen.0020165.st004.pdf]

Table S4: UFW Primers for Gene Set III

SET	Primer	Sequence 5'-3'	Length [bp]	Tm [°C]
3	CG7906_1a	gCCAATCCACAggTCCACTC	20	62.88
3	CG7906_1b	AAACCTggAgTggAccTgTg	20	60.00
3	CG7906_2	gCTCCgTTTgCTgTggTTTC(N)10	30	
3	CG7906_3a	ggTTTgTTCTTTATgTgCTg	20	53.39*
3	CG7906_3b	ggTTTgTTCTTTATgTgCTggA	22	59.13
3	CG7906_4	TAgTTgCggCTTCCgTAgTC	20	60.41
3	CG7906_5	CgACAggATggCTAggAAAT	20	59.15
3	CG7906_6	ggCTggTTATCAggTTCTgg	20	59.55
3	CG7924-1a	CTgggCATTACAACAACTgg	20	59.05
3	CG7924-2a	TTAgggATgCCAggATTTgg(N)10	30	
3	CG7924-3a	gTgATTgggAATgCCAggAT	20	62.03
3	CG7924-4a	CgCACCTTATTATCAgACTT	20	52.22
3	CG7924-5a	TTCCCgTTCCTggTTggTCA	20	65.95
3	CG7924-6a	ggTgACAAgATCAgTTTCggA	21	60.10
3	CG7924-1b	ggTTAgggATgCCAggATTT	20	60.15
3	CG7924-2b	ATCTCTTCCCgTTCCTggTT(N)10	30	
3	CG7924-3b	CTTgTCACCCgCACCTTATT	20	59.99
3	CG7924-4b	AgCAACggCACTTggATAgT	20	59.76
3	CG7924-5b	gCTgTggTgAAgACgAAACA	20	59.88
3	CG7924-6b	CTCCTggACAACATggACCT	20	59.96
3	CG15634-1a	ggAACCAggATAACCgAgTC	20	58.45
3	CG15634-2a	CCACTgTCgTTgCTgATgTT(N)10	30	
3	CG15634-3a	CTgACAACggCTCCACTgTT	20	61.31
3	CG15634-4a	TTggCTTTggTTCTTTTCTA	20	55.27
3	CG15634-5a	CTTgTTTgAATgTCTTgATA	20	48.54
3	CG15634-6a	ATgATggCggATgTgTTTTA	20	58.85
3	CG15634-1b	ATCCgCTgCCAgTATgAgTT	20	59.72
3	CG15634-2b	ATCAAgCCgTCATCgACTTC(N)10	30	
3	CG15634-3b	CATTgACCCggAATTCTACg	20	60.32
3	CG15634-4b	TgACAACggCTCCACTgTT	19	60.30
3	CG15634-5b	CTgTCgTTgCTgATgTTgCT	20	60.06
3	CG15634-6b	ATCgAATggAgAACgCAgAg	20	60.36
3	CG12011-1a	CCCTCCTTCACACCAACCTg	20	62.86
3	CG12011-2a	CCCgCACTTTATTggCAACT(N)10	30	
3	CG12011-3a	AgATTggCCTTgggAgATgA	20	61.90
3	CG12011-4a	gggTTTgATTTCTTgAggTg	20	57.08
3	CG12011-5a	CCATCAgAACACCAAgAAgA	20	56.18
3	CG12011-6a	ACCTCCACgTTgggTTTgAT	20	61.57
3	CG12011-1b	TCTTgACCCTCCTTCACACC	20	60.09
3	CG12011-2b	CTCCACgTTgggTTTgATTT	30	
3	CG12011-3b	gAAgATTggCCTTgggAgAT	20	60.41
3	CG12011-4b	CggAgCCTTgTCCTgTTCTA	20	60.39
3	CG12011-5b	CCAAgAAgAACCAggAgTCg	20	59.84

3	CG12011-6b	CCgCACTTTATTggCAACTT	20	60.13
3	CG4818-1	TCCTTgggTgATTCCgTCTA	20	60.45
3	CG4818-2	TggTTCCgAATAgCCATAgg(N)10	30	
3	CG4818-3a	TgCgggTCTCCTgTTTgCTg	20	66.97
3	CG4818-3b	ggTCTCCTgTTTgCTggACA	20	61.26
3	CG4818-4	CTgACCCAACTCATCCTTgg	20	60.50
3	CG4818-5	ggTTgggCAgTggAAgATTg	20	62.76
3	CG4818-6	ggTCTCCTgTTTgCTggACA	20	61.26
3	Sep5-1a	ggTTgTgCgggCTCggTgTA	20	68.70
3	Sep5-1b	TTgCCTTCAgCTTCACATTg	20	59.99
3	Sep5-2	ggAgCCTggggACTTTggTT(N)10	30	
3	Sep5-3	AgAgTggATTTCCCgAgAg	19	56.79
3	Sep5-4	CACCTCCTgCgTCTTCTTTT	20	59.47
3	Sep5-5	TTTCgCCAgTgCTTTCgTTA	20	61.81
3	Sep5-6	TTCTggACgCTCTTgTTgAC	20	59.01
3	CG10591-1	gCggTCCTggTAAgCCTCTA	20	61.65
3	CG10591-2	gTTCTgATTgCCAggAggTC (N)10	30	
3	CG10591-3	AAggACCTgTgggTggATAg	20	58.87
3	CG10591-4	gACCTggAggTCCTCgTTC	19	59.63
3	CG10591-5	CAAgAgCATTTCACCAACgA	20	59.84
3	CG10591-6	CCCAgTAgCCAgAACCACTg	20	60.70
3	CG10591-7	AAAgCCAAgAgTCCCAAAAA	20	58.81
3	Lcp1-1a	CgAgggCTggTATCCgTTCT	20	63.68
3	Lcp1-1b	ggCTggTATCCgTTCTCATT	20	59.01
3	Lcp1-2	CCTgCTCgATTCCgTTgg (N)10	28	
3	Lcp1-3	CgTACTTAACCTCgACgTgCT	21	59.46
3	Lcp1-4	ggTgTgCAggCTggAATC	18	60.21
3	Lcp1-5	AACATCATCggATTgggAAA	20	60.13
3	Lcp1-6	gTgAgATCCAgCCgAAgTTg	20	60.80
3	Lcp1-7	AAggACATCggCgTggACAT	20	58.81
3	Cg2961-1	CCATAggATCCgTgACCACT	20	59.80
3	Cg2961-2	CCTCCCTgggCATATgAAT (N)10	29	
3	Cg2961-3	CCTgCAgAATgACCTTgATg	20	59.24
3	Cg2961-4	gCCACCACCACCACCAC	17	62.02
3	Cg2961-5	ggCgTggCTATCACATTgTA	20	59.57
3	Cg2961-6	gCgCATACCCATgACCTC	18	60.04
3	Cg2961-7	gCTACCTCCTCCTCCTCCAC	20	60.22
3	slam-1a	TCCTCCCTggTCATCgTAgC	20	62.51
3	slam-1b	CTCCCTggTCATCgTAgCTC	20	59.83
3	slam-2	TTgTgCTTggACTggTTgTT (N)10	30	
3	slam-3	CgCTgAgATCATCACTgTTCA	21	60.00
3	slam-4	CgTggAATTgCTTAggACCA	20	61.02
3	slam-5	TTTgCTgTCTTgTTgTTgACg	21	59.94
3	slam-6	CTgCCgTgTCCACTACCAT	19	59.56
3	slam-7	TTgTAACTgTggCTTTCTgg	20	56.43
3	BcDNA-1	gTggCTgAAgTggTggTATC	20	58.00
3	BcDNA-2	CCTTCAgACAAACgACAAAT (N)10	30	

3	BcDNA-3	CACTCCTCAgCACAgCAATC	20	59.58
3	BcDNA-4	TgATTTTggCACACATTCgT	20	59.97
3	BcDNA-5	TAAgCAgCAgAACggTCCAg	20	61.50
3	BcDNA-6	gCTACCgATAATggAAACCTCT	22	58.67
3	Srp54-1	CAACgggCATACTTCACCTC	20	60.52
3	Srp54-2	ggCAgCTgTCCCTCgAT (N)10	27	
3	Srp54-3	CCgCCACTCgTTCTTAACAT	20	60.13
3	Srp54-4	gTTAATgACTTCgggCggTA	20	59.96
3	Srp54-5	TTCCgTTCTTgAgCATCTCC	20	60.34
3	Srp54-6	ACTTggggTCgTACgTCTTg	20	60.03
3	Srp54-7	gCCAgAACgggTATgACAAT	20	59.82
3	TH1-1a	TATggTgTCCgCCTTCTTgg	20	63.24
3	TH1-1b	ATATggTgTCCgCCTTCTTg	20	59.96
3	TH1-2	CgACTgAAAgTAgCgCTTCA (N)10	30	
3	TH1-3	CCTTgAgATgATTCTCCACCA	21	60.06
3	TH1-4	gTAAATATCCCCggCTCCAT	20	60.01
3	TH1-5	gTgAggAACTTCTCCAggCA	20	60.39
3	TH1-6	ACCACCgCCTTgTAgTTCTC	20	59.21
3	TH1-7	CCAgAgTCgTCgTATTCCAC	20	58.16
3	Bsg25D-1a	TCggAgCCCAggAAgTTgAg	20	64.98
3	Bsg25D-1b	ggAgCCCAggAAgTTgAgTA	20	59.28
3	Bsg25D-2	CCggAgCTCCAgCAgTgA (N)10	28	
3	Bsg25D-3	TTgAACTggCCAAAggACAC	20	61.08
3	Bsg25D-4	CAgAgCTTCAgCAgggACTT	20	59.75
3	Bsg25D-5	TCgTCCAgAAgTCCACACTg	20	59.86
3	Bsg25D-6	gACACgCCCAgCTgATg	17	59.92
3	Bsg25D-7	gCTgCggAACATTTggTAgA	20	61.17
3	CG10154-1	gCACACTTggAgAACAggAT	20	57.71
3	CG10154-2	ggTgAgggCCTAAATgTTCA (N)10	30	
3	CG10154-3	CgATATATTTggAgCAATTTCCA	23	60.17
3	CG10154-4	TggACCgggAgCTACTACAA	20	60.65
3	CG10154-5	TCCgATgAACCATCAAAATg	20	59.33
3	CG10154-6	TAgggCAAATAAACgCCATC	20	59.93
3	CG10154-7a	ATAgTAgTAggAgCCCATTT	20	49.95
3	CG10154-7b	ggAgCCCATTTAATAgCCACT	20	59.48
3	CG7724-1a	ACCgTTTTgTATTCgTATTC	20	51.96
4	CG7724-1b	TTTCgCgTACTCgTTAATgg	20	58.84
3	CG7724-2	ATACATCTACCAgggAgTTT (N)10	30	
3	CG7724-3	AgAgCgCCCAATCCTAATCT	20	60.19
3	CG7724-4	TTgAATgAgATgCAACgAAAA	21	59.29
3	CG7724-5	ATTgCCTAAgCACCCCACTA	20	59.59
3	CG7724-6	TCAACggCCTTTACTTCTCg	20	60.38
3	CG7724-7	gggAAgAgAAgACAACTATT	20	50.08
3	CG2070-1	ACgggCATTCTCgCACTTCT	20	64.42
3	CG2070-2	CggTCACAATCgCCACTCT (N)10	29	
3	CG2070-3	CAAgCCATgTACACggTAgC	20	59.22
3	CG2070-4	TCATTCgTTTTCgTggTgAA	20	60.09
3	CG2070-5	ATAgATTCCgATggCCgAAA	20	61.65

3	CG2070-6	AgCTCCAgAACggTCTCCTT		20	60.39
3	CG2070-7	CAgCgggCAgAACAggTAAT		20	62.46

3	CG6296-1	gCAgCgTgTAAAggTAgAAg		20	55.40
3	CG6296-2	TCCgCCTCCgCCTTACCAAT	(N)10	30	
3	CG6296-3	TAACCgTgTTggTggACAgA		20	60.00
3	CG6296-4	TCTTAggCCACCTTCCCTTT		20	60.07
3	CG6296-5	TCTCgAACCgAAAgTTggTC		20	60.23
3	CG6296-6	gCCTCCTgAgCCTCgTAgTA		20	59.60
3	CG6296-7	CAgCACAAgAAggCACAATC		20	59.45
