# Supplementary material for: Heat-Shock Promoters: Targets for Evolution by P Transposable Elements in Drosophila
Source: PLoS Genet. 2006 Oct 6;2(10):e165. doi: 10.1371/journal.pgen.0020165 (PMC1592238; doi:10.1371/journal.pgen.0020165)
Supplement: Supplementary file 5 [file pgen.0020165.st005.pdf]

**Table S5: Transposable element-specific primers for additional screens**						
			Length	Tm[a]		Ref.
TE	Primer	Sequence 5'- 3'	[bp]	[°C]		
297	297_L1	TTgggAgATgTTTCgAgCAC	20	61.19		
297	297_R1	ATCCCAgCCCAAgTATAggC	20	60.31		
1360	1360_L1	ACggTgAAgggCATATTTTg	20	59.82		
1360	1360_R1	gTATggCCgTTACgCATCTT	20	59.99		
Gypsy	gypsy_L1	CggCTAAATggTATggCAAg	20	60.47		
Gypsy	gypsy_R1	CATCgTgCgTTCACCACTAC	20	60.18		
Jockey	Jockey_L1	gggCAAAAACAACACAgCTT	20	60.15		
Jockey	Jockey_R1	CACCCTCATgACCTCCCTAA	20	59.92		
roo	Roo_L1	AAAggAgCTggCCTTTCTCT	20	59.60		
roo	Roo_R1	gCCCATTgAAATCTTAgTTgC	21	58.71		
I element	I-1R	TAAgCCCCgTAgCTAATgCT	20	59.01		
I element	I-1L	AggTTCgCCTAATTgggATT	20	59.80		
P element	P31IR01	TTCGACGGGACCACCTTAT	19	60.32		
297	297_F_F1	CGCAGCATTCGTTTATCTTTGG	22	63.89	297 F1	b
297	297_F_F2	TGACCTAAGCAGATTTGACTGCC	23	62.83	297 F1	b
297	297_F_F3	CATTCGTTTATCTTTGGCAGCG	22	63.89	297 F	b
297	297_F_F4	AAGCAGATTTGACTGCCCTC	20	59.43	297 F	b
297	297_F_F5	GGTCCAAACCAGCCACTTCC	20	64.04	297 F	b
297	297_F_R1	TTGTATTTCGGGAGAGCCTCGC	22	66.75	297 R1	b
297	297_F_R2	TTGTATTTCGGGAGAGCCTCG	21	63.66	297 R	b
297	297_F_R3	AGGTCAGGCTTTAGGATGTTTACAA	26	62.19	297 R	b
297	297_F_R4	CAGTTGACTAAGGACAACGCC	21	59.79	297 R	b
297	297_F_R5	GTATTTCGGGAGAGCCTCGC	20	63.35	297 R	b
roo	roo_F_F1	TGTTATCTAAGGCGACATGGGTG	23	63.25	Roo F	b
roo	roo_F_F2	ACAGATAAGGTCCTCGCCGC	20	63.82	Roo F	b
roo	roo_F_F3	TGAGTCAGTCACTTGAGATCGTTC	24	60.86	Roo F	b
roo	roo_F_R1	CTAAGGGACTATTTTAGGAGGCGG	24	62.53	Roo R	b
roo	roo_F_R2	CCTAAGTAAATAGTCCCCGCC	21	59.03	Roo R	b
roo	roo_F_R3	AAATAGTCCCCGCCTTATCG	20	60.29	Roo R	b
jockey	jockey_L_l	AAGAAGACTCAAGCGACACC	20	57.1	lower	c
P element	P_L_u	GCCTTCTTTTATCTTTTCTGG	21	55.06	upper	d
Gypsy	gypsy_LP_U	CTATTAAGTCATCCATATAAAGGAA	25	53.22	Upp1_reverse	e
Gypsy	gypsy_LP_L	TTCTATGACTTCTTTCTACTACTCC	25	52.58	Low1_reverse	e

(a) Rozen and Skaletsky (2000) Bioinformatics Methods and Protocols: Methods in Molecular Biology. Humana Press, Totowa, NJ, pp 365-386
(b) Franchini LF, Ganko EW, McDonald JF (2004) Retrotransposon-gene associations are widespread among *D. melanogaster* populations. Mol Biol Evol 21(7): 1323-1331.
(c) Zatsepina OG, Velikodvorskaia VV, Molodtsov VB, Garbuz D, Lerman DN et al. (2001) A *Drosophila melanogaster* strain from sub-equatorial Africa has exceptional thermotolerance but decreased Hsp70 expression. J Exp Biol 204(11): 1869-1881.
(d) Lerman DN, Michalak P, Helin AB, Bettencourt BR, Feder ME (2003) Modification of heat-shock gene expression in *Drosophila melanogaster* populations via transposable elements. Mol Biol Evol 20(1): 135-144.
(e) Lepetit D, Brehm A, Fouillet P, Biemont C (2002) Insertion polymorphism of retrotransposable elements in populations of the insular, endemic species *Drosophila madeirensis*. Mol Ecol 11(3): 347-354.
