# Supplementary material for: Heat-Shock Promoters: Targets for Evolution by P Transposable Elements in Drosophila
Source: PLoS Genet. 2006 Oct 6;2(10):e165. doi: 10.1371/journal.pgen.0020165 (PMC1592238; doi:10.1371/journal.pgen.0020165)
Supplement: Supplementary file 6 [file pgen.0020165.st006.pdf]

Table S6: Primers for intergenic regions						
				Length	Tm	PCR product
	Gene	Primer	Sequence 5'- 3'	[bp]	[°C]	[bp]
1	*Hsp22*	Hsp22_s23	ATGCGATTCGGGAACATATC	20	59.75	2038bp
1		Hsp22_s24	GCCAAAACATCGGTAAGGAA	20	59.94	
1	*Hsp67Ba*	Hsp67Ba_s21	ATTGAAATCGTGCAGCTCCT	20	59.84	2643bp
1	*Hsp23*	Hsp23_s22	CAAGGCTCAACAACAATGGA	20	59.69	
1	*Hsp26*	Hsp26_s1	gCgAAAgCAgAgTAgATAgC	20	54.7	996bp
1		Hsp67Ba_s2	CAAAAACCAAggCgAAAATg	20	60.46	
1	*Hsp27*	Hsp23_s5	TTTgTCCgCCgCTATgCTCTg	21	67.76	2210bp
1		Hsp27_s6	CCTCgTgCTTCCCCTCTACCA	21	65.89	
1	*Hsp68*	Hsp68_s27	GCGTTGAATTGCTTCAGAGG	20	60.91	1553bp
1		Hsp68_s28	GGTACGGTTACCCTGGTCGT	20	61.05	
1	*Hsp70Aa***	Hsp70Aa_s11	CATCCCAAAAATCTgTAAAgC	21	56.44	1429bp
1		Hsp70Ab_s12	ACTgTgTTTCTggggTTCAT	20	56.9	
1	*Hsp70Aa*	70AaF01	GCAGGCATTGTGTGTGAGTT	20	59.76	1264bp
1		70AaR02	GCCCCTTTGAGTTGTAACCAT	21	60.24	
1	*Hsp70Ab*	70AbF03	CGGCACCATAAGCATAACCT	20	59.98	846bp
1		70AbR04	TCATGTCCTCTGCGATCTTG	20	59.94	
1	*Hsp83*	CG14965_s9	TTAggTgTTgCTgCTTTggAg	21	60.43	2856bp
1		Hsp83_s10	CATCgTTgTTCTTggAggTgA	21	61.08	
1	*HsrOmega*	HsrO_s31	ATTCGCGTCTGAATGAGGAC	20	60.23	2810bp
1		HsrO_s32	GTACTGCTGCTGCTCGTCTG	20	59.95	
1	*DnaJ-1*	DnaJ-1_s33	GTTCCGTTCGGCTGTTTTAC	20	59.61	1475bp
1		DnaJ-1_s34	CGAGGCCCAGAATCTTGTAG	20	59.83	
1	*DnaJ-60*	DnaJ-60_s13	gCTCTTTCTCCTTgTTTTT	19	51.33	2484bp
1		DnaJ-60_s14	TAgTCCCCCgATgATgTgC	19	61.87	
1	*DnaJ*	DnaJ_s35	CAGCTGGAGCAGGTAGATCC	20	59.97	622bp
1		DnaJ_3	TGGAGAGGATTCGGTAGGAG	20	59.23	
1	*CG9920*	CG9920_s36	TGATCGGTGTCCAAGTCAAC	20	59.53	2827bp
1		CG9920_5	CCTCGAAACGCTGGATTAGA	20	60.34	
1	*Hsp60*	Hsp60_s15	AgCgTAATCAgCgAAAATg	19	56.59	2185bp
1		Hsp60_s16	ggAgCgAgCAAgCgAAACT	19	63.05	
1	*Hsp60b*	Hsp60b_s29	GATCCAGGAATTTCGTCTGC	20	59.63	1661bp
1		Hsp60b_s30	ATCATCATTGCCCGTACTCC	20	59.78	
1	*Hsc70-2*	Hsc70-2_s19	TATgCgCCCATgACTATgAg	20	59.67	1409bp
1		Hsc70-2_s20	TCTCCACCTTgCTgTTCTgC	20	61.55	

1	*Hsc70-3*	Hsc70-3_s41	ACTCAAGTGCCAGGATACGG	20	60.13	2879bp	
1		Hsc70-3_s38	CACCTACCACTGACGTGTCG	20	60.22		
1	*Hsc70-4*	Hsc70-4_7	CTGAAGGTCCCCGAGTATCC	20	60.84	2402bp	
1		Hsc70-4_s39	ATCGGTGAAGGCAACATAGG	20	59.96		
1	*Hsc70-5*	Hsc70_5-7	GATTGGTCCGCTGCTGTAGA	20	61.35	1732bp	
1		Hsc70-5_s40	AGTTGGTTGTTCCCAGATCG	20	59.97		
1	*Hsp67Ba*	Hsp67Ba_s21	ATTGAAATCGTGCAGCTCCT	20	59.84	2643bp	
1	*Hsp23*	Hsp23_s22	CAAGGCTCAACAACAATGGA	20	59.69		
2	*Gapdh1*	Gapdh1_s42	TCTTCTGGCCTCTGGAGTTG	20	60.52	835bp	
2		Gapdh1_1	TCCGTTAATTCCGATCTTCG	20	60.03		
2	*βTub56Da*	βTub56Da_s43	AGGGAAATCGTTCACATCCA	20	60.3	2129bp	
2		βTub56Da_s44	CACCCATGTACGCCATCATA	20	60.2		
2	*βTub56Db*	βTub56Db_s45	GGCCACAAAGAGAATAGCACA	20	60.26	1298bp	
2		βTub56Db_s46	CTGACCAGCTTGGATGTGAA	20	59.83		
2	*Adh*	Adh_s47	GTCTGGACACCAGCAAGGAG	20	60.9	1708bp	
2		Adh_s48	TGGGGATTTTCCGTACTCTG	20	59.9		
2	*Sgs4*	Sgs4_s49	CAACCAGGGTAACGAAAAGC	20	59.6	954bp	
2		Sgs4_4	ACTCCAAGCGCATCTTGACT	20	60		
2	*Su(s)*	Su(s)_7	CGATAAATTGCACTCGCTGA	20	60	3018bp	
2		Su(s)_s50	GAAGATTGCTGCTCGTCCTC	20	60.1		
2	*Act5C*	Act5C_s51	GGATACTCCTCCCGACACAA	20	59.9	1998bp	
2		Act5C_4	AGGATACCACGCTTGCTCTG	20	60.4		
2	*RpL32*	RpL32_s52	GAATGCGTTTTGTGTGTTGG	20	60	2054bp	
2		RpL32_6	CTCTGTTGTCGATACCCTTGG	21	59.8		
2	*Elf*	Elf_a_s53	GCGATCCCTGGGTATGTAGA	20	60.8	1461bp	
2		Elf_a_s54	ACTGGGCACAAATTCTACCG	20	59.2		
2	*Elf*	Elf_b_s55	CCACATCATCCTCCAATTCC	20	60.1	2601bp	
2		Elf_b_s56	CGTGAACTCAGCATCTTCCA	20	60		
2	*CG32061*	CG31061_7	CACATCGCTACTTCGTTCCT	20	58	1117bp	
2		CG8040_s61	TATGGCCTTAACCAGCATCC	20	59.9		
2	*CG4750*	CG4750_s60	TTTTCCGTAAGCCCAATGTC	20	59.9	2951bp	
2		CG4750_5	CACAACACCCTTGGGACTCT	20	60		
2	*αTub84B_*	αTub84B_S57	GTACTGCCTTTCTGCGTTGG	20	60.1	1484bp	
2		αTub84B_1	CTCGCTGAAGAAGGTGTTGA	20	60		
2	*αTub84D*	αTub84D_s58	CCCGTGAAGACGATCAAAGT	20	60.1	1919bp	

2		αTub84D_6	GTTCCAGTACGGACCTCGTC	20	59.6	
2	*cup*	cup_s59	GATCACCCTTCTGCCAAAGT	20	59.1	595bp
2		Cup_7	AATTCGATCGCCTCCTCTTT	20	60.2	
3	*CG7906*	CG7906_s62	TGAGCTATGCACCAAGTTCG	20	60.01	2900bp
3		CG7906_1	GCCAATCCACAGGTCCACTC	20	62.88	
3	*CG7924*	CG7924_s63	AGGCCTTCCAGGAAAAGGAG	20	61.95	2086bp
3		CG7924_3	GTGATTGGGAATGCCAGGAT	20	62.03	
3	*CG15634*	CG15634_s64	ACATTCCCACGTTCTGTGAG	20	58.57	1008bp
3		CG15634_1	GGAACCAGGATAACCGAGTC	20	58.45	
3	*Sep5*	Sep5_s65	GGCTCCGTCTCGATGACTAC	20	59.83	1345bp
3		Sep5_6	TTCTGGACGCTCTTGTTGAC	20	59.01	
3	*CG10591*	CG10591_s66	CGGCCTTCTACTTTGGCTAC	20	59	2397bp
3		CG10591_5	CAAGAGCATTTCACCAACGA	20	59.84	
3	*slam*	slam_s67	CGACCAGTACCATGACTCC	20	56.35	3561bp
3		slam_7	TTGTAACTGTGGCTTTCTGG	20	56.43	
3	*Srp54*	Srp54_s68	GTCACTATCGGCCATCACCT	20	59.96	1224bp
3		Srp54_6	ACTTGGGGTCGTACGTCTTG	20	60.03	
3	*TH1*	TH1_s69	CTGTACACGCCGGAAAAGTT	20	60.2	760bp
3		TH1_4	GTAAATATCCCCGGCTCCAT	20	60	
3	*Bsg25D*	Bsg25D_s70	CTGCTTGTCGTTCAGGAAGC	20	61.12	1347bp
3		Bsg25D_7	GCTGCGGAACATTTGGTAGA	20	61.17	
3	*CG10154*	CG10154_s71	CCACATTGGAGCAGACATTG	20	60.11	1198bp
3		CG10154-4	TGGACCGGGAGCTACTACAA	20	60.65	
3	*CG7724*	CG7724_6	TCAACGGCCTTTACTTCTCG	20	60.38	1885bp
3		CG7724_s72	TTGCTTGATGAGGTGCTGAC	20	59.99	
3	*CG2070*	CG2070_s73	GAAGTTCTGCGCCTTGCTGT	20	62.95	1787bp
3		CG2070_7	CAGCGGGCAGAACAGGTAAT	20	62.46	
3	*CG6296*	CG6296_s74	CACAGCCGCTCAGTGATCTA	20	60.16	831bp
3		CG6296_5	TCTCGAACCGAAAGTTGGTC	20	60.23	

*Bettencourt BR, Feder ME (2002) Rapid concerted evolution via gene conversion at the Drosophila hsp70 genes. J Mol Evol 54(5): 569-586.
