# Supplementary material for: Heat-Shock Promoters: Targets for Evolution by P Transposable Elements in Drosophila
Source: PLoS Genet. 2006 Oct 6;2(10):e165. doi: 10.1371/journal.pgen.0020165 (PMC1592238; doi:10.1371/journal.pgen.0020165)

Table S7: P element-specific primers for orientation and length

Primer	Sequence 5'- 3'	Length [bp]	Tm [°C]
P82L	ATTCAGTGCACGTTTGCTTG	20	59.91
P192L	CTgCAAAgCTgTgACTggAg	20	59.77
P225L	CgTgCCgAAgTgTgCTATTA	20	59.90
P574L	ggAgTACgAAATgCgTCgTT	20	60.14
P774L	AAggATTTCCTTTgCCCAgT	20	59.94
P879L	AgTTgATgACgCCgACAAg	19	59.85
P1899R	AACTgCAgTggAgTgggATg	20	61.13
P2181R	gCCAgCTTTCAgAgTTgTCC	20	60.00
P2321	CGGAGGATGCGATGGAAT	18	61.97
P2419R	ATAAgTCCgCCgTgAgACAC	20	60.14
P2457R	CATTCTAgCTCTTTTAACTTCTCTTgA	27	58.23
P2549R	ACgTTTCgCgCTgCTAATA	19	59.62
P2833R	gCTTgCAATAAgTgCgAgTg	20	59.64

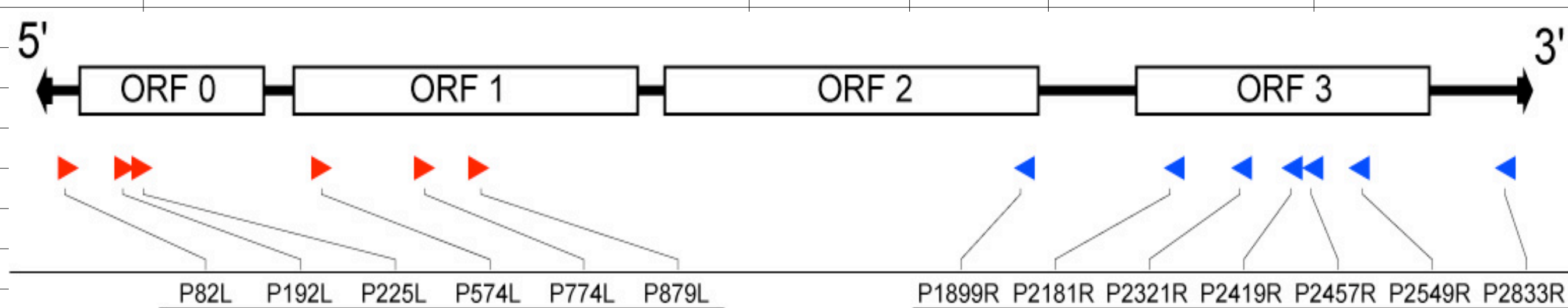

Supplement: Supplementary file 7 [file pgen.0020165.st007.pdf]
